# *Alpinia oxyphylla* Miq.: Chemical Constituents, Neuroprotective and Metabolic Effects, and Translational Challenges—A Review of Preclinical Evidence

**DOI:** 10.1002/fsn3.72553

**Published:** 2026-11-01

**Authors:** Ziyan Yang, Hemei Yuan, Dengyou Nie, Jun Sheng, Titi Liu, Huanhuan Xu

**Affiliations:** ^1^ College of Science Yunnan Agricultural University Kunming China; ^2^ Key Laboratory of Development and Utilization of Food and Medicinal Resources, Ministry of Education Yunnan Agricultural University Kunming China; ^3^ College of Food Science and Technology Yunnan Agricultural University Kunming China

**Keywords:** *Alpinia Oxyphylla* Miq, chemical constituents, metabolic regulation, neuroprotection, PI3K/Akt pathway

## Abstract

*Alpinia oxyphylla* Miq. (AOM), a traditional resource with both medicinal and edible uses, has attracted increasing interest for its reported neuroprotective and metabolic activities. However, its active constituents, molecular mechanisms, and potential applications in functional food development remain incompletely defined. This review consolidates evidence on the major bioactive constituents of AOM, including diphenylheptanoids, polysaccharides, flavonoids, terpenoids, and phenolic acids, and summarizes reported molecular targets and pathways associated with neural and metabolic homeostasis. The available preclinical evidence suggests that AOM and its constituents may affect experimental models of Alzheimer's disease, Parkinson's disease, diabetes, and related complications through modulation of neuroinflammation, oxidative stress, PI3K/Akt‐associated signaling, and the gut microbiota‐metabolite axis. Some AOM‐containing herb‐pair formulations have also shown enhanced effects in preclinical models, and AOM constituents have been associated with anti‐allergic, cardioprotective, and antitumor activities. Finally, this review discusses translational challenges for AOM‐based functional food and nutraceutical development, including bioavailability, quality control, delivery systems, safety, and the scarcity of human clinical evidence. Overall, the current evidence supports AOM as a promising botanical resource for further mechanistic and translational research, while therapeutic and product‐development claims should be interpreted with appropriate caution until supported by robust clinical and safety data.

## Graphical abstract

*Alpinia oxyphylla* Miq. (AOM), a medicinal and edible resource, contains diphenylheptanoids, polysaccharides, flavonoids, terpenoids, and phenolic acids. These constituents exert multi‐target synergistic effects by modulating neuroinflammation, oxidative stress, and the PI3K/Akt pathway, thereby improving Alzheimer's disease, Parkinson's disease, and depression, while also regulating glucose and lipid metabolism, protecting the liver and kidney, alleviating hyperuricemia, and exerting cardioprotective, antitumor, and anti‐allergic activities, supporting AOM‐based functional food development pending clinical validation.

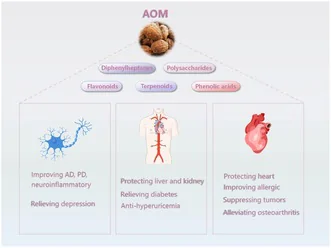

## 1 Introduction

*Alpinia oxyphylla* Miq. (AOM), commonly known as “Yi Zhi Ren”, is the dried mature fruit of *Alpinia oxyphylla*, a perennial herbaceous plant belonging to the Zingiberaceae family (Zhang et al. 2018). It is mainly cultivated in Guangdong, Hainan, Fujian, and Yunnan provinces, with its chemical composition and biological properties significantly influenced by the growing environment (Pan et al. 2023). Characterized by an aromatic, pungent, and slightly bitter flavor, AOM serves as both a traditional Chinese medicine (TCM) and a food ingredient, reflecting the deep‐rooted “food‐medicine homology” concept in Chinese culinary and medicinal traditions. The dried ripe fruit, known as Yi Zhi Ren in Chinese, is officially recognized as a food‐medicine dual‐use substance in China (Wei et al. 2025; Zhang et al. 2018). Traditional culinary applications of the fruit include preserved fruits, candied snacks, medicinal wines, congee, herbal soups, and teas (Wei et al. 2025). The Chinese Pharmacopeia recommends a daily dosage of 3–10 g of the dried mature fruit (Wei et al. 2025; Zhang et al. 2018).

AOM boasts a well‐documented history of medicinal use, firmly established in foundational TCM texts such as Shennong's Classic of Materia Medica and recognized in the current edition of the Chinese Pharmacopoeia (Zhang et al. 2018). It is widely employed in TCM, both as a monotherapy and as part of polyherbal formulations, where specific herb‐pair combinations have shown enhanced effects in preclinical studies (Qi, Cheng, Gong, et al. 2020; Qi, Cheng, Jing, et al. 2020; Qi, Jing, Cheng, et al. 2020). Modern phytochemical studies have identified a wealth of bioactive constituents in AOM, including diphenylheptanes, terpenoids, flavonoids, polysaccharides, volatile oils, and steroids (Liao and Zhao 2024; Zhang et al. 2018). AOM exhibits significant efficacy in improving cognitive impairment and regulating glucose and lipid metabolism (He et al. 2019; Sun et al. 2023; Xie et al. 2017, 2018). However, current research remains largely focused on crude extracts, leaving the material basis of its health benefits, constituent‐specific molecular mechanisms, and the translation of its traditional dietary use into scientifically supported modern functional foods as critical challenges yet to be systematically addressed.

Therefore, this review aims to systematically summarize the chemical constituents of AOM and integrate evidence across its reported molecular targets and pathways, with a particular emphasis on the interplay between neural and metabolic regulation. By integrating recent advances in neuroprotection, metabolic modulation, herb‐combination studies, and other bioactivities, we seek to construct a comprehensive framework for understanding the broader biological effects of AOM. Furthermore, this review will propose targeted strategies and viable pathways for translating these mechanistic insights into practical applications under the concept of “medicinal and edible homology”. Ultimately, our goal is to provide a scientific foundation for the development and high‐value utilization of AOM as a traditional resource.

Recent reviews have covered chemical constituents, pharmacology, pharmacokinetics (Liao and Zhao 2024), neuroprotective effects and mechanisms (Wei et al. 2025), and integrated phytochemical, pharmacological, bibliometric, and patent perspectives (Chen et al. 2026), but largely follow a descriptive, compound‐by‐compound approach. This review instead proposes the PI3K/Akt pathway as an integrating node, incorporates herb‐pair interactions and the gut microbiota‐metabolite axis, and extends to translational barriers and methodological caveats in functional food development, shifting from a composition–activity inventory to a composition‐evidence‐network‐translation framework.

Notably, studies have further expanded the evidence base for AOM, particularly through chromosome‐level genomic and multi‐omics analyses of geo‐herbalism, integrated transcriptome‐metabolome characterization of quality‐associated biomarkers, microbiome‐and formulation‐oriented pharmacological investigations, and emerging food‐application studies (Pan et al. 2023, 2025). These advances are integrated into the relevant sections below; however, human clinical evidence remains very limited.

## 2 Methods

The present review follows the fundamental principles of systematic literature synthesis, aiming to comprehensively delineate the phytochemical and pharmacological advances of *Alpinia oxyphylla* Miq. and to establish a logically coherent narrative framework. The literature search was conducted without strict time restrictions, thereby covering both historical and recent publications. The databases searched included PubMed, Web of Science Core Collection, and CNKI, with English search terms set as “*Alpinia oxyphylla*”, “*Alpinia oxyphylla* Miq.”, and “*Alpinia oxyphylla* fruit”. To ensure quality and relevance, predefined inclusion and exclusion criteria were applied: studies were required to explicitly address the isolation, identification, or quantitative determination of chemical constituents from *A. oxyphylla* , or to investigate the biological activities of its extracts or purified compounds; original research articles and systematic reviews were considered. A two‐phase inductive approach was then used for data organization. Phase I comprised thematic classification, whereby all articles were primarily grouped into two major axes, phytochemical constituents and biological activities. Phase II involved comparative analysis and logical framework construction, including assessment of reported structural differences, methodological reliability, and evidence linking specific components to observed activities. These analyses informed the overarching logical thread and the order of secondary discussion.

## 3 Chemical Components

AOM contains multiple bioactive compounds that exhibit therapeutic potential against a range of diseases. Various extraction methods have been employed to isolate these constituents, including aqueous extraction (Dong et al. 2023) as well as solvent‐based techniques utilizing ethanol, ethyl acetate, or petroleum ether (Li, Xiao, et al. 2021). Pharmacochemical chemistry studies have shown that the main constituents of AOM are terpenes, diphenylheptane, and flavonoids (Li et al. 2013), as well as catechins, protocatechuic aldehyde, (−)‐epicatechol, kaempferol, geraniol, baicalein, and many sesquiterpenes such as guaiane, chicory alkane, menthol, chaibutane, and so forth (Qiu et al. 2023). However, current research remains largely focused on crude extracts, with insufficient attention paid to the specific biological roles of individual purified constituents. Future studies should expand upon the currently fragmentary isolation of pure compounds, structure–activity relationship (SAR) analyses, and target validation to clarify the mechanistic underpinnings of AOM's bioactivity. This is particularly critical for its application in functional foods, where standardized and well‐characterized components are essential for ensuring both efficacy and safety.

### 3.1 Diphenylheptanes

Diphenylheptanes, which are recognized for their distinctive structural frameworks and diverse biological activities, represent a major class of natural products. These compounds are widely distributed in various plants, especially within the Zingiberaceae family, with well‐known examples including curcumin, turmerone, and gingerols. AOM, a medicinally significant species within this family, has been reported to contain substantial amounts of diphenylheptanoids (Wang et al. 2015).

Among the diphenylheptanoids isolated from AOM, yakuchinone A and yakuchinone B (Figure 1) are notable for their significant biological activities, including well‐documented anti‐inflammatory and antitumor properties (Chun et al. 2002). Future studies should aim to comprehensively evaluate their pharmacological potential, with emphasis on specific molecular targets and mechanisms of action. Furthermore, interdisciplinary research integrating food science and nutrition is encouraged to explore their applications in functional food development.

**FIGURE 1:**
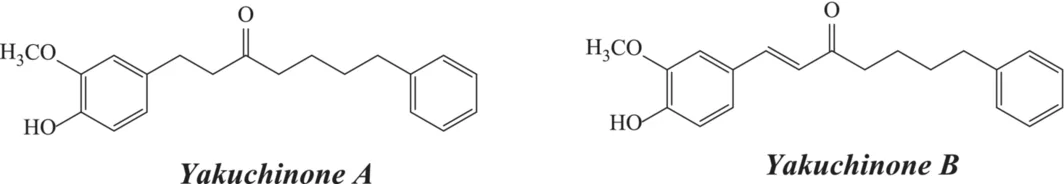
Chemical structures of representative diphenylheptanoids isolated from AOM. The figure shows the characteristic diarylheptanoid skeleton and the structures of yakuchinone A and yakuchinone B, the major bioactive constituents of this class with reported anti‐inflammatory and antitumor activities.

### 3.2 Polysaccharides

*Alpinia oxyphylla* Miq. polysaccharides (AOMPs) are a class of complex, multifunctional polymers with significant biological activity. A novel polysaccharide, AOP70‐2‐1, has been isolated from AOMP and exhibits anti‐neuroinflammatory activity (Shi et al. 2020). It consists of β‐D‐GlcAp‐(1→, →2,3,6)‐α‐D‐Galp‐1→, α‐L‐Araf‐(1→, →2,5)‐α‐L‐Araf‐(1→, →4)‐α‐D‐Glcp‐(1→, →3,4)‐α‐D‐Xylp‐(1→, →3,6)‐β‐D‐Manp‐1→, and α‐L‐Rhap‐1→. Using hot water, hydrochloric acid, and alkali to treat AOM, it was found that the main components of the extract were different for different treatment methods. The extract treated with hot water and hydrochloric acid was mainly composed of glucose, while the extract treated with alkali was rich in arabinose (Wang et al. 2024). It is worth noting that polysaccharides extracted by alkaline treatment exhibit excellent antioxidant capacity, indicating their potential applications in nutritional supplements and treatments for oxidative stress. In contrast, the polysaccharide extracted by hydrochloric acid treatment showed stronger inhibitory activity on α‐amylase, highlighting its prospects in the treatment of hyperglycemia and diabetes. The selection of extraction solvents significantly influences the yield, molecular weight, surface morphology, monosaccharide composition, and crystalline structure of AOMPs, underscoring its critical role in polysaccharide isolation.

In a purification study, AOMPs were fractionated via sequential chromatography using DEAE‐52 cellulose and Sephadex G‐100 columns, yielding three purified fractions: AOMP1, AOMP2, and AOMP3. Structural analysis revealed that AOMP1 consists of arabinose, galactose, glucose, xylose, mannose, galacturonic acid, and glucuronic acid in a defined molar ratio. Critically, AOMP1 demonstrates dual immunomodulatory effects: augmentation of both Th1 and Th2 responses and potent activation of antigen‐specific immunity (Yang et al. 2020). The biological activity of AOMP has not yet been thoroughly investigated, signifying the potential for further exploration into its pharmacological value. This aspect represents a significant opportunity in the field of natural product research. Despite these promising findings, the biological activities of AOMP fractions remain insufficiently explored, indicating a substantial need for further pharmacological investigation.

### 3.3 Flavonoids

Flavonoids derived from AOM exhibit confirmed antioxidant, anti‐inflammatory and neuroprotective properties, with kaempferol, quercetin, and naringenin serving as representative constituents. In relevant studies, some related flavonoids have been identified from the *Alpinia oxyphylla* Miq. extract (AOE) (Figure 2A). A phytochemical investigation isolated a novel flavonol glucuronide, kaempferol 3‐O‐[2″,3″‐O‐diacetyl‐6″‐methyl ester]‐β‐D‐glucuronopyranoside, from AOE (Figure 2B). This compound showed weak in vitro inhibition against U251 cells (IC_50_ = 128.51 μM). In addition, two compounds, ferulic acid and methyl gallate, were identified (Zhao et al. 2024). Current research on bioactive constituents of AOM prioritizes tectochrysin (He et al. 2019), a structurally distinctive flavonoid conferring multifarious biological activities (Figure 2C). Studies have established that tectochrysin isolated from AOM exhibits significant antitumor efficacy against prostate, colorectal, and breast carcinomas (Patel 2023). Additionally, tectochrysin has been reported to ameliorate allergic airway inflammation by suppressing Th2 responses and oxidative stress (Fang et al. 2021).

**FIGURE 2:**
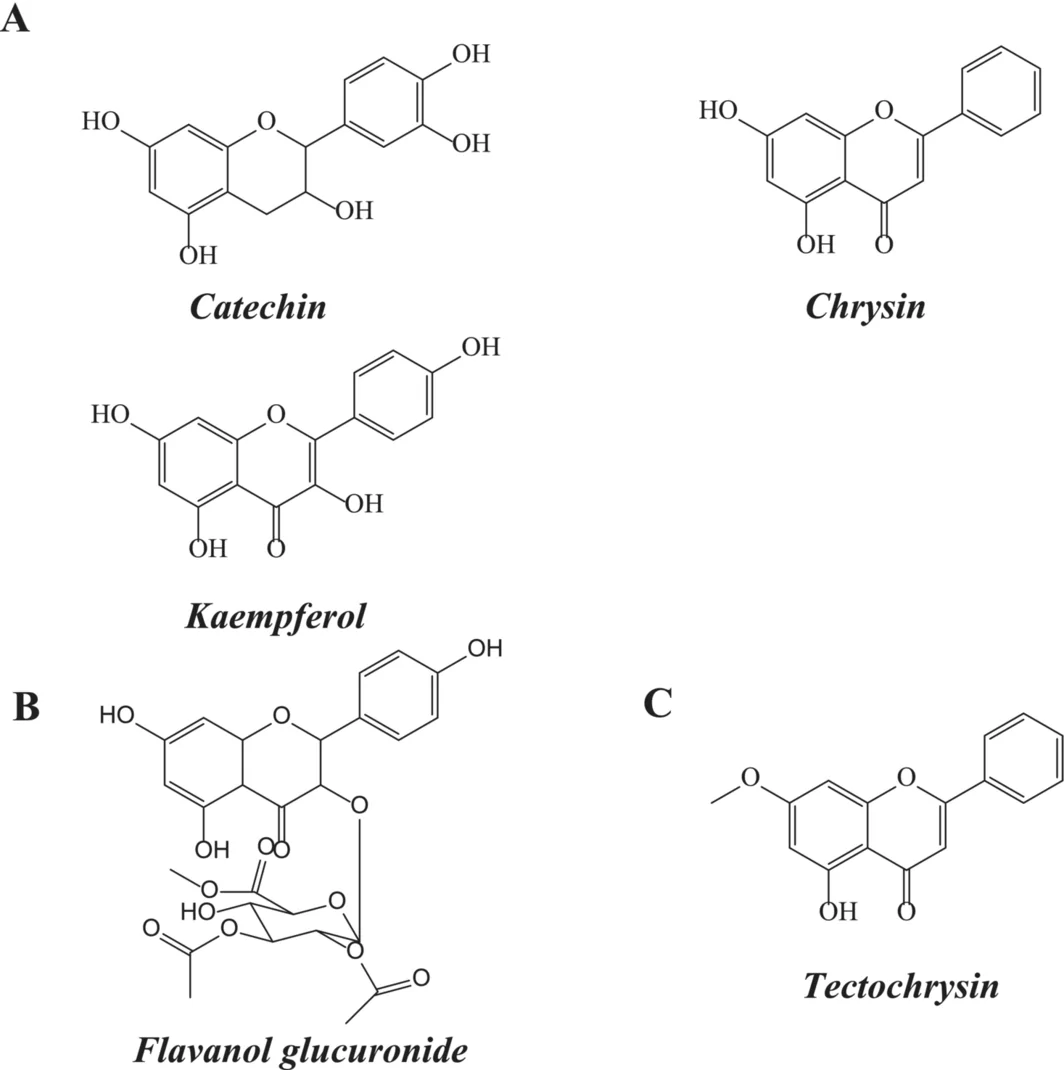
Chemical structures of representative flavonoids identified in AOM. The figure displays the structures of catechin, chrysin, kaempferol, tectochrysin, and a novel flavonol glucuronide (kaempferol 3‐O‐[2″,3″‐O‐diacetyl‐6″‐methyl ester]‐β‐D‐glucuronopyranoside). These flavonoids contribute to antioxidant, anti‐inflammatory, and neuroprotective effects.

### 3.4 Terpenoids

AOM contains a variety of terpenoids, each exhibiting distinct biological activities (Cui et al. 2023). To date, researchers have isolated and identified more than 90 structurally diverse sesquiterpenes from AOM (Zhang et al. 2018). A significant proportion of these sesquiterpenes have been shown to possess a wide range of biological activities (Dong et al. 2023), including neuroprotection (He et al. 2018; Wang et al. 2018), antioxidant, anti‐inflammatory (Qiu et al. 2023), antibacterial (Wang et al. 2015), and antitumor properties. This group has garnered significant attention due to its unique characteristics and extensive bioactivities, indicating broad research prospects. GC–MS analysis of its volatile compounds identified the following compounds as the main components, including L‐β‐pinene, trans‐sabinene hydrate, cyclohexene, and (E)‐labba‐8(17),12‐dien‐15,16‐dialdehyde (Peng et al. 2022). Several terpenoids have been isolated from AOM (Qiu et al. 2023; Zhu et al. 2024), and it has been demonstrated that some of these compounds possess significant neuroprotective and preventive effects against diseases such as renal fibrosis (Figure 3).

**FIGURE 3:**
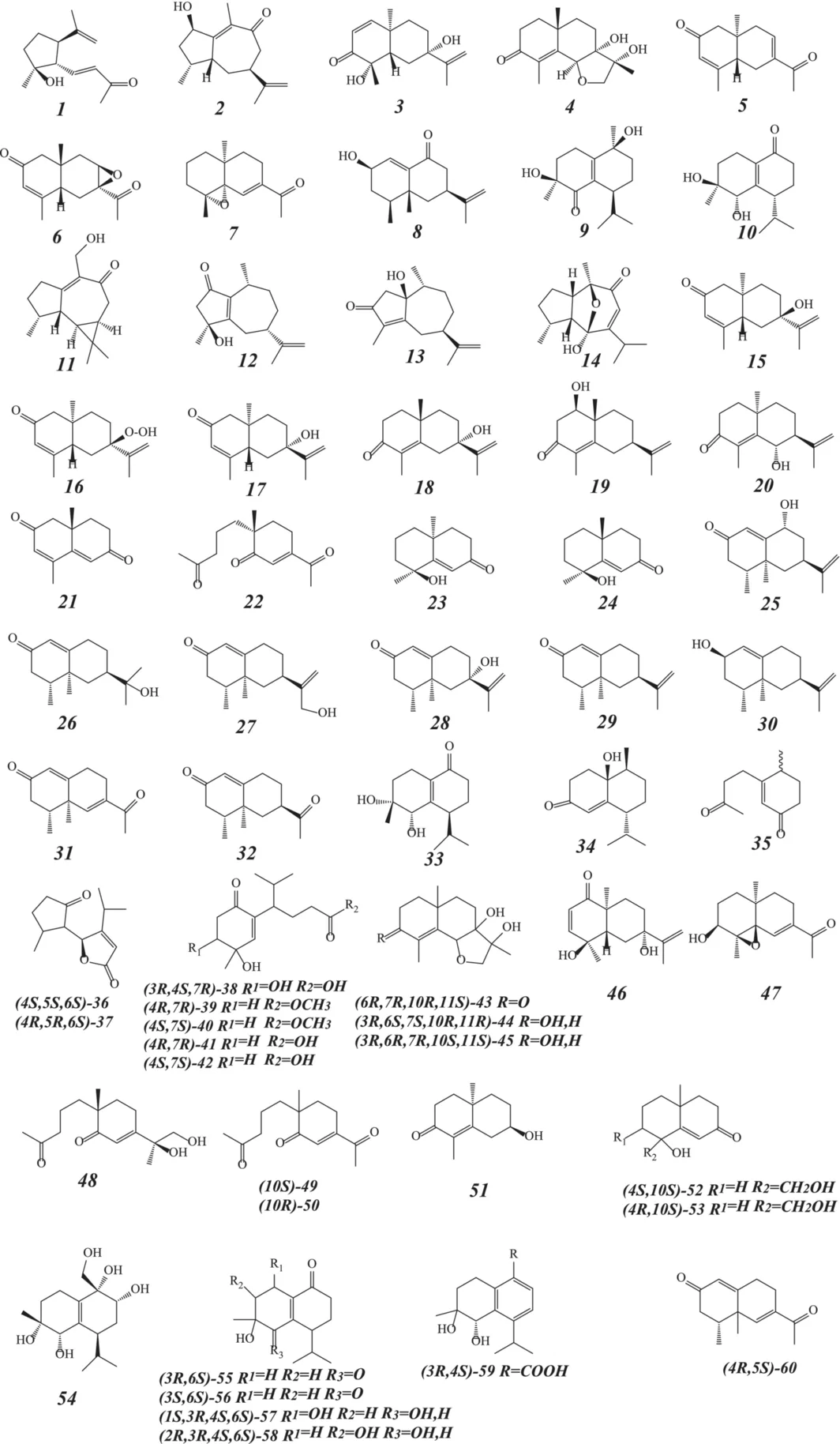
Chemical structures of representative terpenoids (part 1) from AOM.

Nootkatone, the principal component of *Alpinia oxyphylla* volatile oil (AOVO), is of particular interest due to its diverse biological activities (Dong et al. 2023). A study isolated and identified two undescribed sesquiterpene compounds from AOM. These were found to be one demethyl‐eucalyptane type (compound 1) and one guaiacolane type (compound 2), as well as two known analogues (compound 3–4) (Figure 4A). Compound 1 was obtained as a yellow oil with the molecular formula C_14_H_20_O_2_, while compound 2 was characterized as a yellow amorphous solid (C_15_H_22_O_3_) containing five degrees of unsaturation. The two known compounds were identified as alpinenone (3) (Bai et al. 2025). Moreover, investigations have identified five novel terpenoids: oxyphyllenodiol B, (4S*,5E,10R*)‐7‐oxo‐tri‐nor‐eudesm‐5‐en‐4β‐ol, 7‐epi‐teucrenone, (+)‐(4R,5S,7R)‐13‐hydroxynootkatone, (E)‐labda‐12,14‐dien‐15(16)‐olide‐17‐oic acid (Zuo et al. 2021). Furthermore, isolation studies yielded 17 new sesquiterpenes and 23 known analogues from AOM (Figure 4C), among which compound 20 (Figure 4C) significantly suppressed protein (COX‐2, iNOS) and mRNA (TNF‐α, IL‐6, COX‐2, iNOS) expression levels (Dong et al. 2023). Another investigation identified 35 sesquiterpenes, including 10 previously unreported compounds, some of which enhanced the viability of H_2_O_2_‐induced SH‐SY5Y cells, supporting their neuroprotective potential (Qiu et al. 2023). The isolation of 48 compounds has been reported, including 24 new sesquiterpenes that exhibit renal protective activity (Zhu et al. 2024). These findings significantly enrich the chemical database of AOM‐derived terpenoids; however, systematic studies to elucidate their structure–activity relationships and mechanisms of action are still warranted.

**FIGURE 4:**
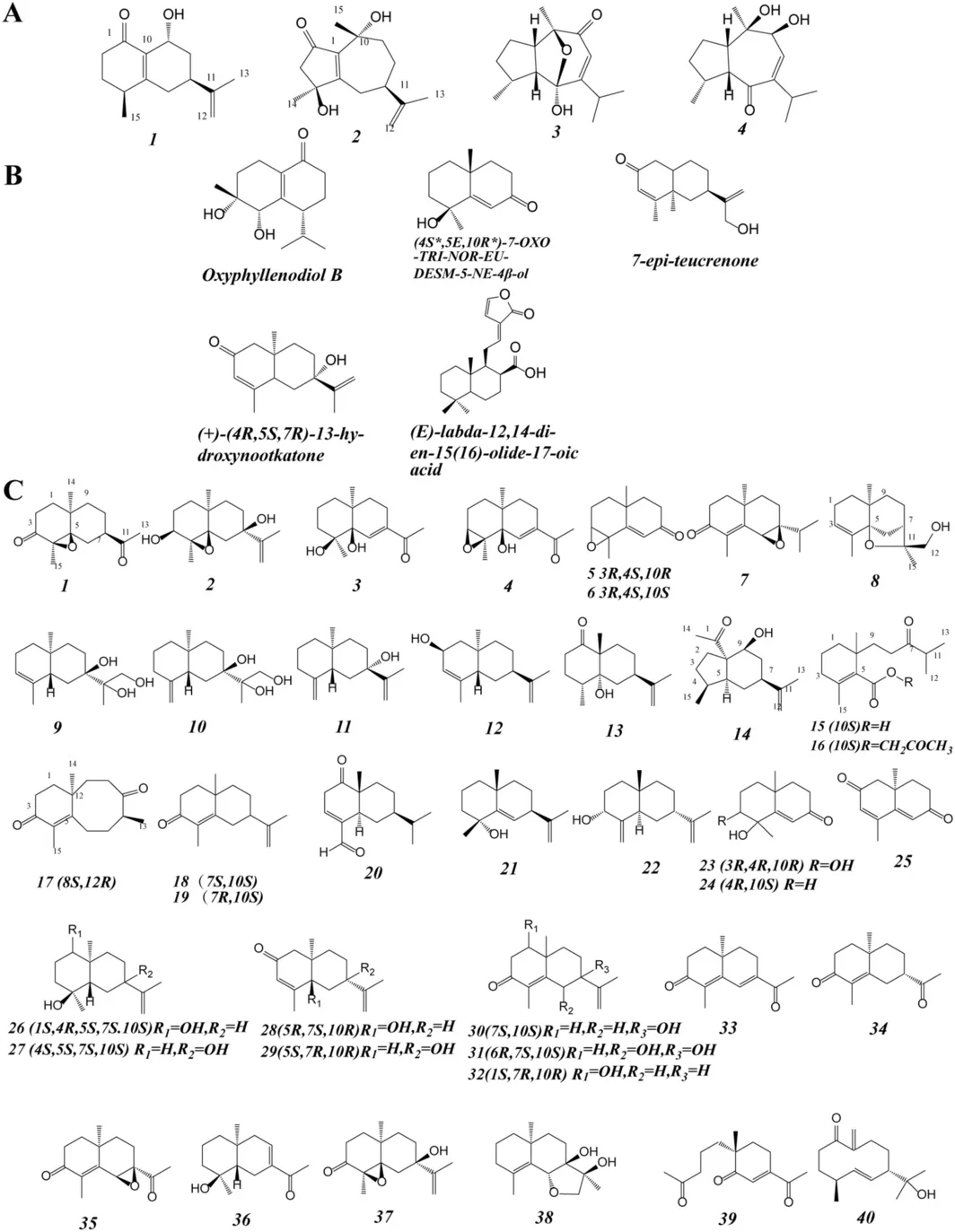
Chemical structures of representative terpenoids (part 2) from AOM.

### 3.5 Phenolic Acids

Research has shown that AOM contains a variety of phenolic acid compounds (He et al. 2020), such as protocatechuic acid (PCA), protocatechuic aldehyde, and p‐coumaric acid (Figure 5). Phenolic acid is a major bioactive component in AOM, which has the effects of improving cognitive impairment (He et al. 2020) and anti‐inflammatory (Cheng et al. 2021). Chromatographic analysis revealed PCA content of 0.064 mg/g in AOE via HPLC quantification (Cheng et al. 2021), while p‐coumaric acid (P‐CA) was identified at 4.6 mg/g in subsequent research (He et al. 2020). Research has demonstrated that AOM contains Alpinia PCA, which has been shown to possess certain neuroprotective properties (Shi et al. 2006). Given the significant neuroprotective effects of phenolic acids such as PCA and P‐CA, future studies should prioritize elucidating their combined or complementary mechanisms in neuroprotection. Unraveling these mechanisms will not only provide a theoretical foundation for the clinical utility of AOM phenolic acids as natural neuroprotective agents but also offer a novel research paradigm for the design of multi‐component neuroprotective strategies. To facilitate rapid overview of the major bioactive constituents discussed above, the representative compounds, their chemical classes, primary activities, and associated disease targets are summarized in (Table 1).

**FIGURE 5:**
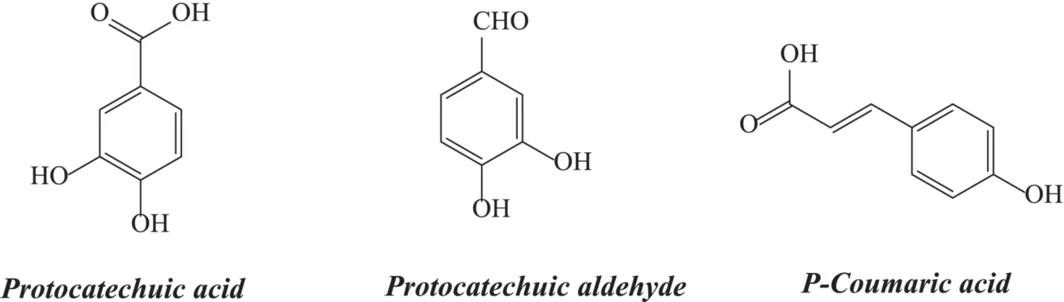
Chemical structures of representative phenolic acids from AOM. The figure shows protocatechuic acid, protocatechuic aldehyde, and p‐coumaric acid, which exhibit antioxidant, anti‐inflammatory, and neuroprotective properties.

**TABLE 1:** Chemical classes, major activities, disease targets/models, evidence levels, and references of representative bioactive constituents from AOM.

Compound	Class	Major activity	Disease‐target/model	Evidence level	References
Yakuchinone A	Diphenylheptanoid	Anti‐inflammatory, antitumor	Skin tumor models	In vivo	Chun et al. (2002)
Yakuchinone B	Diphenylheptanoid	Anti‐inflammatory, neuroprotective	Neurodegenerative dementia	In vitro, in vivo	Zeng et al. (2022)
AOP70‐2‐1	Polysaccharide	Anti‐neuroinflammatory	Alzheimer's disease (AD)	In vivo	Shi et al. (2020)
Tectochrysin	Flavonoid	Antitumor, anti‐inflammatory	Prostate, colorectal, and breast cancer	In vitro, in vivo	Park et al. (2016); Park et al. (2015); Patel (2023)
Kaempferol‐3‐O‐[2″,3″‐O‐diacetyl‐6″‐methyl‐ester]‐β‐D‐glucuronopyranoside	Flavonoid	Antitumor (weak)	U251 glioblastoma cells	In vitro	Zhao et al. (2024)
Nootkatone	Sesquiterpenoid	Neuroprotective, anti‐inflammatory, renoprotective	AD, diabetic nephropathy	In vitro, in vivo	Chen et al. (2021); He et al. (2018); Yong et al. (2022)
Oxyphyll‐A	Sesquiterpenoid	Neuroprotective, antioxidant	AD, Parkinson's disease (PD)	In vitro, in vivo	Bian et al. (2021); Shao et al. (2024)
Compound 20 (eudesmane‐type) (Figure 4C)	Sesquiterpenoid	Anti‐inflammatory	LPS‐induced inflammation	In vitro	Dong et al. (2023)
Protocatechuic acid (PCA)	Phenolic acid	Neuroprotective, antioxidant	AD, PD	In vitro, in vivo	Guan et al. (2006); He et al. (2020); Shi et al. (2006)
p‐Coumaric acid (P‐CA)	Phenolic acid	Neuroprotective, promotes neurogenesis	Cerebral ischemia	In vivo	He et al. (2020)

### 3.6 Structure–Activity Relationship Insights

Although a comprehensive SAR analysis for AOM constituents is currently constrained by the limited number of systematic structure–activity studies, several preliminary trends can be discerned from the available data. For diarylheptanoids, synthetic analogue studies of yakuchinones have revealed that the optimal linker length between the two aryl groups and the presence of an α, β‐unsaturated enone moiety are essential for nitric oxide (NO) inhibitory activity in LPS‐activated macrophages; the most potent synthetic analogue achieved an IC_50_ of 0.92 μM, significantly surpassing the parent compounds (Lee et al. 2006). The phenolic hydroxyl groups on the aromatic rings also contribute to radical scavenging activity, as demonstrated by the DPPH assays (Bian et al. 2013). For sesquiterpenoids, structural diversity plays a critical role in anti‐inflammatory potency. Among 40 structurally diverse eudesmane‐type sesquiterpenoids isolated from AOM, compounds with rearranged bicyclic skeletons or rare 5/6‐ and 6/8‐fused ring systems exhibited moderate to strong inhibition of NO, TNF‐α, and IL‐6 production in LPS‐stimulated BV‐2 cells (Dong et al. 2023). Notably, compound 20 (Figure 4C) significantly suppressed COX‐2 and iNOS protein expression as well as the mRNA levels of TNF‐α, IL‐6, COX‐2, and iNOS (Dong et al. 2023). Synthetic analogues of yakuchinone B have also been explored for anti‐arthritic activity. A benzylideneacetophenone derivative (JC3) inhibited carrageenan/kaolin‐induced arthritis in rats and suppressed inflammatory cytokine expression in IL‐1β‐stimulated human fibroblast‐like synoviocytes (Sur et al. 2019). These findings suggest that structural modification of AOM‐derived diarylheptanoids may generate candidates with altered or enhanced anti‐inflammatory profiles. For polysaccharides, structure–function relationships are strongly influenced by extraction methods: alkaline extraction yields polysaccharides with distinct monosaccharide composition and superior antioxidant capacity, while acid extraction favors α‐amylase inhibitory activity (Wang et al. 2024). For flavonoids, SAR analysis identified that a 7‐methoxy substitution is slightly favorable for ABCG2 inhibition in tectochrysin, although 6‐prenylation produces a much stronger effect (Ahmed‐Belkacem et al. 2005).

Despite these emerging insights, it must be acknowledged that current SAR knowledge for AOM constituents remains fragmentary and largely qualitative. Systematic quantitative SAR (QSAR) studies, comprehensive evaluation of substitution effects across congeneric series, and target‐specific SAR profiling are largely absent from the literature. Future investigations combining rational chemical synthesis, systematic structural modification, and standardized bioactivity assays are urgently needed to establish robust SAR frameworks that could guide the rational design of AOM‐derived lead compounds.

## 4 AOM Modulates Neural and Metabolic Homeostasis Across Reported Pathways

### 4.1 Cross‐Domain Neuroprotection: Convergent Mechanisms Across Experimental Models

Neurological disorders such as AD, neuroinflammation, depression, and PD display distinct clinical symptoms yet share common pathological mechanisms, including oxidative stress, neuroinflammation, apoptotic signaling, and dysregulation of neurotrophic pathways. AOE and their bioactive components have shown promise in ameliorating AD (He et al. 2019), modulating PD progression (Li et al. 2016), alleviating neuroinflammation (Qiu et al. 2023; Xu et al. 2020), and exerting antidepressant effects (Qi et al. 2024), indicating activity across multiple disease‐relevant processes rather than a single established target. Moreover, several neuroprotective compounds, including yakuchinone B, chrysin, and yakuchinone A, have been identified from AOM (Zeng et al. 2022), providing a molecular basis for its pleiotropic pharmacological activities.

#### 4.1.1 Coordinated Modulation of Neuroinflammation and Oxidative Stress

AD is a neurodegenerative disorder characterized by progressive cognitive decline, often accompanied by muscle atrophy and motor deficits. Its core pathological hallmarks include the presence of senile plaques composed of abnormally deposited amyloid‐β (Aβ) and neurofibrillary tangles (NFTs) formed by hyperphosphorylated tau protein (Breijyeh and Karaman 2020; Schirinzi et al. 2018). In cerebral ischemia–reperfusion (I/R) injury, release of multiple pro‐inflammatory mediators exacerbates the progression of cerebral infarction (Fang et al. 2016). Microglia and astrocytes play central roles in the production of these pro‐inflammatory factors. Pharmacological inhibition of TLR4‐mediated activation of these glial cells in the ischemic region has been shown to effectively alleviate cerebral infarction symptoms (Zhang, Song, et al. 2020).

AOM also alters metabolic profiles in preclinical AD models. It improves learning and memory deficits in AD rat models by modulating amino acid, lipid, and energy metabolism (Sun et al. 2023). Untargeted metabolomics analysis further reveals that AOM significantly normalizes the levels of 15 plasma metabolites, primarily enriched in sphingolipid, glycerophospholipid, fatty acid, and bile acid metabolism pathways (Zhou et al. 2022).

In terms of neuroinflammation suppression, the polysaccharide AOP70 has been shown to restore serum levels of pro‐inflammatory mediators, including NO, IL‐1β, PGE2, and TNF‐α, in AD mice, thereby improving cognitive function (Shi et al. 2020). Similarly, nootkatone significantly ameliorates neurobehavioral performance in AD mice by reducing the oxidative stress marker malondialdehyde (MDA) and inhibiting acetylcholinesterase (AChE) activity (He et al. 2018).

Protocatechuic acid (PCA), a representative neuroprotective component of AOM, markedly suppresses MPP^+^‐induced apoptosis in PC12 cells in PD‐related models. This protective effect is closely associated with its ability to improve mitochondrial membrane permeability and alleviate oxidative stress damage (Guan et al. 2006). In an H_2_O_2_‐induced oxidative injury model in PC12 cells, PCA not only effectively inhibits cell death but also reduces lipid peroxidation and significantly enhances the activities of glutathione peroxidase (GPx) and superoxide dismutase (SOD) in aged rats, leading to improved cognitive function (Shi et al. 2006). More importantly, in both AD and cerebral ischemia models, PCA treatment promotes neurological recovery and neural stem cell (NSC) proliferation, enhances spatial learning and memory consolidation, and reduces anxiety‐like behaviors (He et al. 2020).

Notably, PCA and chrysin exhibit synergistic neuroprotective effects when administered in combination (Zhang et al. 2015). Chrysin enhances the protective efficacy of PCA, increasing the survival of 6‐hydroxydopamine‐treated PC12 cells and reducing lactate dehydrogenase release. This combination significantly attenuates chemically induced dopaminergic neuron damage in both zebrafish and mouse PD models (Zhang et al. 2015). Mechanistically, the co‐treatment upregulates the transcriptional activity of Nrf2, thereby promoting the expression of antioxidant enzymes such as HO‐1 and SOD, while concurrently suppressing the activation of NF‐κB and the expression of iNOS (Figure 6). Collectively, these findings indicate that PCA exerts broad prospects in intervening neurodegenerative pathologies through the coordinated regulation of anti‐apoptotic, antioxidant, and pro‐neurogenic mechanisms.

**FIGURE 6:**
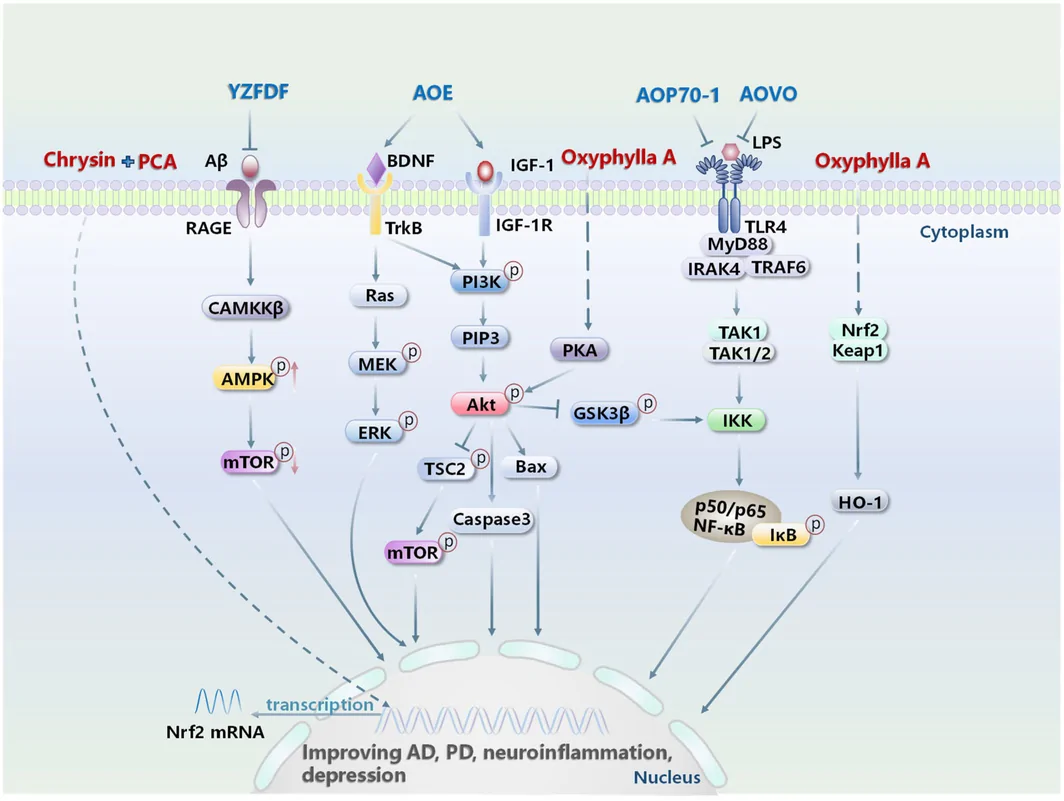
Proposed neuroprotective network of AOM centered on the PI3K/Akt signaling hub. Arrows indicate activation or positive regulation; T‐bars indicate inhibition. Akt, protein kinase B; AMPK, adenosine monophosphate‐activated protein kinase; AOE, *Alpinia oxyphylla* Miq. extract; Aβ, amyloid β‐protein; BAX, Bcl‐2 associated X‐protein; BDNF, brain‐derived neurotrophic factor; CAMKKβ, Ca^2+^/Calmodulin‐dependent protein kinase kinase beta; Caspase3, cysteine‐dependent aspartate‐directed Protease 3; ERK, extracellular – signal‐regulated kinase; GSK‐3β, glycogen synthase kinase‐3β; HO‐1, heme oxygenase‐1; IGF‐1, insulin‐like growth factor 1; IGF‐1R, insulin‐like growth factor 1 receptor; IKB, inhibitor of nuclear factor–κB; IKK, IκB kinase; IRAK4, interleukin‐1 receptor‐associated kinase 4; eap1, kelch‐like ECH‐associated protein 1; LPS, lipopolysaccharide; MEK, mitogen‐activated protein kinase; mTOR, mechanistic target of rapamycin; MyD88, myeloid differentiation primary response protein 88; NF‐κB, nuclear factor‐kappa B; Nrf2, nuclear factor erythroid 2‐related factor 2; P50, nuclear factor‐κB p50 subunit; P65, nuclear factor‐κB p65 subunit; PI3K, phosphatidylinositol 3‐kinase; PIP3, phosphatidylinositol 3,4,5‐trisphosphate; PKA, protein kinase A; RAGE, receptor for advanced glycation end‐products; Ras, rat sarcoma virus oncogene homolog; TAK1, transforming growth factor‐β‐activated kinase 1; TAK1/2, transforming growth factor‐β‐activated kinase 1/2; TLR4, toll‐like receptor 4; TRAF6, TNF receptor‐associated factor 6; TrkB, tropomyosin‐related kinase B; TSC2, tuberous sclerosis complex 2.

#### 4.1.2 The PI3K/Akt Pathway as a Central Hub in AOM‐Mediated Neuroprotection

Accumulating evidence establishes the PI3K/Akt signaling pathway as a central regulator of cell survival, proliferation, metabolism, and autophagy, playing a pivotal role in maintaining homeostasis in the nervous system. This review proposes that the PI3K/Akt pathway serves as a critical hub for the broad neuroprotective effects of AOM. It integrates multiple protective processes, including autophagy regulation, inflammation suppression, neurotrophic support, and antioxidant defense, through extensive cross‐talk with key pathways such as RAGE, TLR4, BDNF/TrkB, and Nrf2, thereby forming a coordinated regulatory network (Figure 6).

In AD, AOM targets Aβ toxicity through multiple complementary mechanisms that converge on this hub. It improves cognitive function by activating the PI3K/Akt pathway to mediate anti‐apoptotic effects (Li et al. 2022). Additionally, *Oxyphylla A* exerts antioxidant effects via the Akt/GSK3β and Nrf2/Keap1/HO‐1 pathways (Bian et al. 2021). Akt not only enhances cell survival by inhibiting GSK3β but also reinforces antioxidant defenses via Nrf2 activation, demonstrating positive synergy between these pathways (Li, Du, et al. 2021).

In cerebral ischemia/reperfusion (I/R) injury, the anti‐inflammatory effects of AOM are closely linked to this central hub. AOE downregulates key pro‐inflammatory mediators, including TLR4 and NF‐κB (Cheng et al. 2021). Notably, the arabinoxylan AOP70‐1, isolated from AOM, directly inhibits LPS‐induced TLR4/MyD88/NF‐κB activation (Chang et al. 2024). Since the PI3K/Akt pathway has been shown to negatively regulate NF‐κB activity, AOE likely restrains NF‐κB‐driven neuroinflammation through a dual mechanism: direct inhibition (e.g., by AOP70‐1) and indirect suppression via Akt‐mediated negative feedback. Following I/R injury, AOM activates the BDNF/TrkB/Akt pathway, promoting hippocampal neurogenesis (He et al. 2020). As BDNF/TrkB serves as a canonical activator of PI3K/Akt, this establishes a positive feedback loop that links neurotrophic support with anti‐inflammatory and regenerative processes. Moreover, AOM significantly activates the IGF‐1‐mediated PI3K/Akt pathway, enhancing the proliferation and survival of RSC96 Schwann cells (Chang et al. 2017).

In depression models, this hub functions as a nexus connecting neurotrophic and anti‐inflammatory signaling. The antidepressant‐like effects of AOM depend entirely on TrkB receptor activation and involve downstream stimulation of the CREB/BDNF signaling system through both PI3K/Akt and MAPK/ERK pathways (Yan et al. 2016). These findings indicate that both PI3K/Akt and MAPK/ERK contribute downstream of TrkB to pathways associated with neural plasticity. Concurrently, AOVO ameliorates depression‐like behaviors by inhibiting the TLR4/MyD88/NF‐κB pathway (Wu et al. 2023), further underscoring how AOM indirectly suppresses neuroinflammation via this central signaling hub to achieve coordinated therapeutic outcomes.

In PD models, the mechanism of *Oxyphylla A* illustrates a profound integration of PI3K/Akt signaling with antioxidant response and mitochondrial biogenesis pathways. It activates the PKA/Akt/GSK3β and CREB/PGC‐1α/NRF‐1/TFAM signaling cascades (Shao et al. 2024). This not only promotes neuronal survival by inhibiting GSK3β but also addresses core pathologies of PD by driving mitochondrial biogenesis and enhancing antioxidant defense through Nrf2 activation, supporting an integrated protective response.

Importantly, the above compilation of evidence, while strongly suggestive of a central role for the PI3K/Akt axis, warrants a clear distinction between experimentally established facts and our proposed integrative model. Direct causal evidence—derived from targeted pharmacological inhibition or genetic ablation of PI3K/Akt components—is currently available only for a subset of AOM constituents, such as *Oxyphyll A* and total flavonoids in AD/PD models. In contrast, the convergence of autophagy regulation (RAGE/CAMKKβ/AMPK/mTOR), neurotrophic support (BDNF/TrkB), and anti‐inflammatory signaling (TLR4/NF‐κB) onto a single PI3K/Akt hub remains, at present, a hypothetical working model based on pathway cross‐talk extrapolation rather than validated mechanistic integration. Accordingly, we propose this network model as a testable framework to guide future investigations, wherein direct target engagement, isoform‐specific contributions, and dynamic signaling kinetics require rigorous experimental dissection.

### 4.2 Regulation of Metabolic Homeostasis by AOM Across Multiple Processes

AOM exhibits significant metabolic regulatory activity, affecting several metabolic processes, particularly in the regulation of glucose and lipid metabolism, renal protection, and uric acid homeostasis (Figure 7).

**FIGURE 7:**
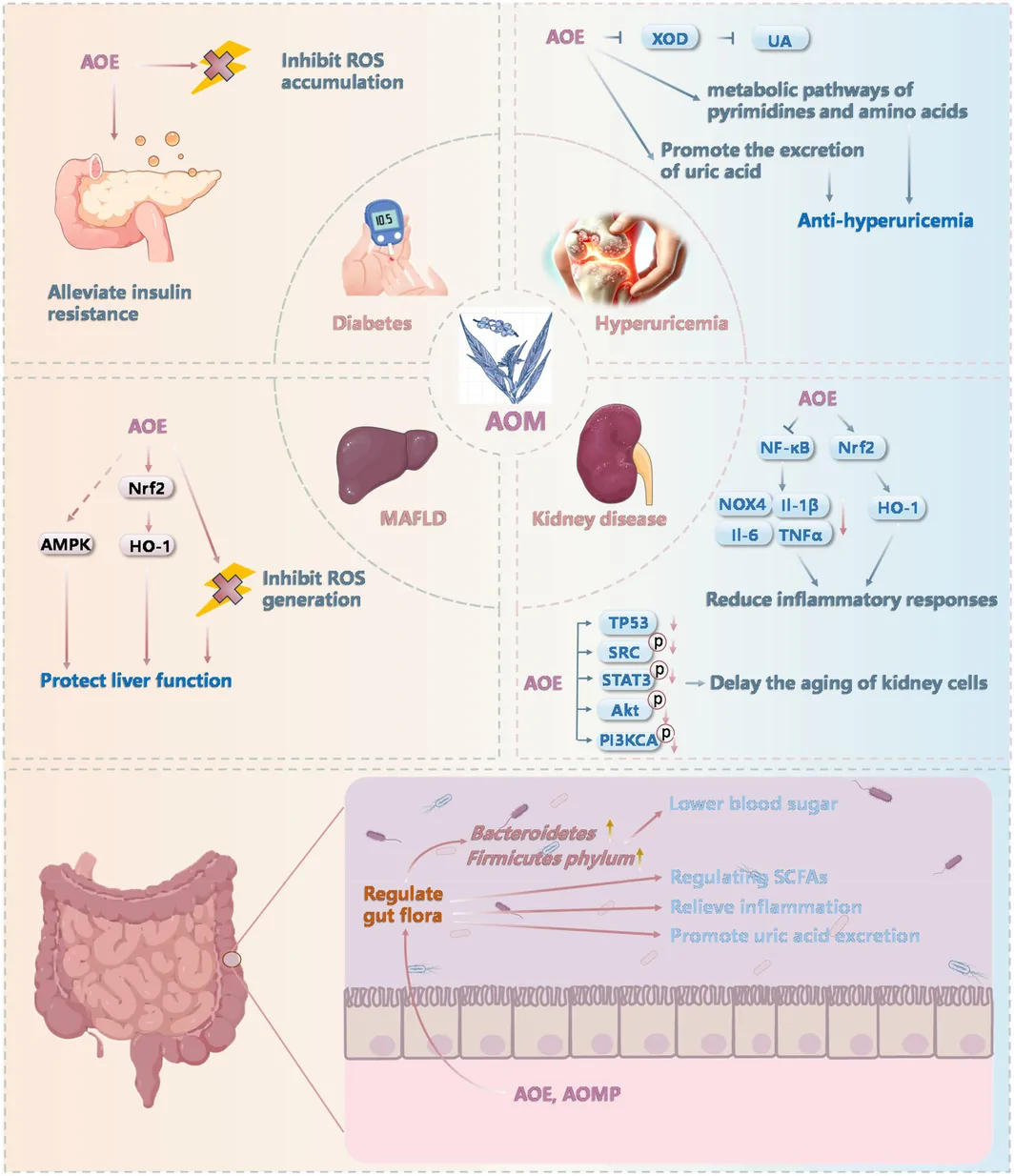
Multi‐pathway regulation of metabolic homeostasis by AOM. The schematic summarizes the principal mechanisms by which AOM extracts, polysaccharides, and monomeric compounds modulate glucose and lipid metabolism, renal protection, and uric acid homeostasis. Key pathways include AMPK activation, Nrf2/HO‐1 antioxidant signaling, inhibition of NF‐κB‐mediated inflammation, and regulation of gut microbiota‐derived metabolites. Arrows denote promotion or activation; T‐bars denote inhibition. AKT, AKT serine/threonine kinase; AOE, *Alpinia oxyphylla* Miq. extract; AOM, *Alpinia oxyphylla* Miq.; AOMP, *Alpinia oxyphylla* Miq. polysaccharides; IL‐1β, interleukin‐1β; IL‐6, interleukin‐6; iNOS, inducible nitric oxide synthase; MAFLD, metabolic‐associated fatty liver disease; NOX4, NADPH oxidase 4; PI3KCA, phosphatidylinositol‐3‐kinase catalytic alpha polypeptide; ROS, reactive oxygen species; SRC, v‐src avian sarcoma (Schmidt‐ruppin A‐2) viral oncogene homolog; STAT3, signal transducer and activator of transcription 3; TNF‐α, tumor necrosis factor‐α; TP53, tumor protein 53; XOD, xanthine oxidase.

AOE possesses marked anti‐type 2 diabetic properties, effectively reducing blood glucose levels in a dose‐dependent manner without affecting body weight (Du et al. 2017; Xie et al. 2017). Its glucose‐lowering mechanisms involve the inhibition of reactive oxygen species (ROS) accumulation, improvement of insulin sensitivity, and modulation of specific miRNA expressions that influence diabetes‐related signaling pathways. Notably, characteristic diarylheptanoids and sesquiterpenoids in AOE alleviate type 2 diabetes‐associated cognitive dysfunction by inhibiting BACE1 activity and reversing Aβ‐induced neuronal damage (Ge et al. 2024).

In diabetic nephropathy models, AOE ameliorates renal function by regulating oxidative stress and PTEN expression (Xie et al. 2017). Its primary active component, nootkatone, reduces renal oxidative stress damage, inflammatory cell infiltration, and apoptosis, while improving pathological structures and decreasing the expression of fibrosis markers (Chen et al. 2021). Advanced nano‐delivery systems have further enhanced the bioavailability and therapeutic efficacy of nootkatone. For instance, AOM‐derived nootkatone‐loaded nanoparticles demonstrate good biocompatibility, promote macrophage M2 polarization, alleviate renal inflammation, and facilitate tissue repair (Deng et al. 2025). At the cellular level, sesquiterpenes derived from AOM significantly suppress TGF‐β1‐induced extracellular matrix component accumulation and α‐smooth muscle actin expression, and downregulate fibronectin and type I collagen expression in a concentration‐dependent manner (Zhu et al. 2024). Furthermore, flavonoid constituents in AOM also exhibit anti‐fibrotic effects on renal tubular cells, with some components showing superior efficacy to nootkatone in inhibiting fibronectin expression (Zhu et al. 2023).

In terms of hepatoprotection, nootkatone demonstrates substantial improvement in metabolic‐associated fatty liver disease (MAFLD). It corrects high‐fat diet‐induced glucose and lipid metabolic disorders, improves glucose tolerance, reduces hepatic lipid accumulation and steatosis, and thereby preserves liver function (Yong et al. 2022). This mechanism may be associated with the activation of the AMPK signaling pathway. Additionally, both the crude ethanol extract and dichloromethane fraction of AOM exhibit protective effects against CCl_4_‐induced hepatotoxicity in vitro and in vivo, involving multiple pathways such as free radical scavenging, inhibition of ROS generation, reduction of lipid peroxidation, and activation of the Nrf2 pathway (Zhang et al. 2017).

AOE also demonstrates anti‐hyperuricemic activity. Its combination with the conventional drug allopurinol enhances the therapeutic effect, normalizing serum uric acid levels and inhibiting xanthine oxidase (XOD) activity in both serum and the liver (Sung and Kim 2022). AOMP inhibits uric acid synthesis by regulating pyrimidine and amino acid metabolism pathways, while also promoting renal uric acid excretion and inhibiting its reabsorption, thereby exerting uric acid‐lowering effects (Ren et al. 2024).

Gut microbiota plays a critical role in the metabolic regulation mediated by AOE. AOE modulates the gut microbiota structure in db‐/db‐ mice, increasing the relative abundance of Bacteroidetes and Firmicutes, which contributes to blood glucose reduction (Xie et al. 2018). It also increases gut bacterial diversity, regulates short‐chain fatty acid levels in diabetic nephropathy rats, and alleviates inflammatory responses (An et al. 2023). In addition, AOMP promotes uric acid excretion by modulating the gut microbiota, thereby achieving anti‐hyperuricemic effects (Ren et al. 2024). However, causal microbial mediators, reproducibility across extraction protocols, and human validation remain insufficiently established.

At the molecular level, nootkatone alleviates renal oxidative stress and inflammatory responses by inhibiting caspase‐9, caspase‐3, and the NF‐κB pathway, downregulating NOX4, IL‐1β, IL‐6, and TNF‐α expression, while upregulating Nrf2 and HO‐1 expression and activating the Nrf2/HO‐1 pathway (Dai et al. 2023). AOE also ameliorates renal pathological injury and delays renal cell senescence by downregulating TP53 levels and the phosphorylation of SRC, STAT3, PIK3CA, and AKT (Yan et al. 2024). Network pharmacology analysis suggests that AOM may exert therapeutic effects in hyperuricemia‐associated immune and inflammatory diseases by interacting with targets such as PPARG, ESR1, and PTGS2. Among its constituents, stigmasterol acts as a core active component, significantly upregulating ESR1 and PPARG expression (Zhang et al. 2024).

In summary, preclinical studies suggest that AOM may modulate multiple metabolic processes through several mechanisms, including glucose and lipid metabolism, hepatic and renal function, uric acid homeostasis, and gut microecology. These findings provide a basis for further investigation, although clinical validation is needed to support the development of natural product‐based intervention strategies for metabolic diseases.

### 4.3 Preclinical Evidence for Combinations of AOM With Other Medicinal Plants

Research indicates that herb pairs combining AOM with other medicinal plants can produce enhanced or complementary effects in several preclinical disease models. The AOM‐Schisandra chinensis herb pair (ASHP) significantly ameliorates Aβ‐induced cognitive dysfunction, biochemical abnormalities, and histopathological damage in mice (Qi et al. 2019). Its neuroprotective mechanisms involve suppression of the NF‐κB inflammatory signaling pathway and reduction of apoptosis. Further studies demonstrate that ASHP modulates the PI3K/Akt/GSK3β/CREB signaling axis, reducing β‐amyloid deposition and attenuating tau hyperphosphorylation, thereby reversing cognitive deficits and neurodegenerative pathology (Qi, Jing, Cheng, et al. 2020). At the compositional level, the combination of nootkatone from AOM and schisandrin (S‐CH) from Schisandra chinensis activates the PI3K/AKT/GSK‐3β/mTOR pathway, significantly downregulating key inflammatory factors including NF‐κB, IKK, IL‐1β, IL‐6, and TNF‐α, consequently inhibiting apoptosis and excessive autophagy and effectively mitigating neuroinflammation (Qi, Cheng, Gong, et al. 2020).

Studies on another herb pair, AOM‐Acorus tatarinowii (AOAT), show that its essential oil components improve behavioral performance in AD model mice, reduce hippocampal neuronal loss, increase brain acetylcholine levels, and decrease γ‐aminobutyric acid and glutamate concentrations. In cellular models, this essential oil protects human cerebral microvascular endothelial cells (hCMEC/D3) from Aβ‐induced damage, potentially through modulation of RAGE, LRP1, GLUT1, and GLUT3 protein expression (Li 2021). Furthermore, the Yizhi Formula Daifang (YZFDF) suppresses autophagy via the RAGE/CAMKKβ/AMPK/mTOR pathway, in which AMPK acts as an mTOR inhibitor, thereby effectively restoring tight junction protein integrity and reducing Aβ deposition in the hippocampus (Chan et al. 2020). This mechanism converges with the canonical PI3K/Akt/mTOR pathway, in which Akt activates mTOR to inhibit autophagy, illustrating how AOM employs distinct upstream signals to precisely modulate the “double‐edged sword” of autophagy for neuronal protection.

Meanwhile, the AOM‐Schizonepeta tenuifolia herb pair (AOST) alleviates skin inflammation in allergic dermatitis model mice by regulating Th1/Th2 balance and reducing pro‐inflammatory cytokine levels, suggesting involvement of the gut microbiome‐skin axis (Zhang, Qiu, et al. 2020).

In summary, through scientifically designed combinations with various medicinal plants, AOM may engage multiple pathways across different experimental models, demonstrating broad application prospects in neuroprotection, metabolic regulation, and immune balance.

### 4.4 Other Potential Bioactivities

AOM effectively inhibits angiotensin II (Ang II)‐induced H9C2 cell apoptosis and reverses Ang II‐induced mitochondrial membrane potential collapse, indicating its role in preserving mitochondrial integrity and counteracting cardiomyocyte apoptosis (Chang et al. 2013). Additionally, AOE regulates collagen synthesis and degradation pathways, helping maintain collagen homeostasis and reducing the risk of myocardial fibrosis (Chang et al. 2019). In aging heart models, AOM enhances stem cell repair capacity. Pretreatment with AOM alleviates doxorubicin (DOX)‐induced mitochondrial apoptosis and DNA damage in H9C2 cells. Furthermore, combined application of AOM with adipose‐derived mesenchymal stem cells (ADMSCs) significantly mitigates age‐related structural and functional decline in cardiac tissue. This occurs through activation of the Nrf2 antioxidant pathway and suppression of overall oxidative stress, thereby optimizing the ADMSC microenvironment and enhancing their paracrine repair potential (Chang et al. 2021; Lin et al. 2021).

Nootkatone exhibits antitumor effects by activating the ATF4‐CHOP‐CHAC1 signaling pathway, inhibiting proliferation and migration of glioblastoma multiforme (GBM) cells (Wang et al. 2025). Tectochrysin, when combined with cetuximab (an anti‐EGFR monoclonal antibody), synergistically inhibits colon cancer cell growth through multiple mechanisms: reducing IL‐8 mRNA expression, decreasing COX‐2 levels, blocking EGFR signal transduction, and suppressing NF‐κB and AP‐1 transcriptional activity (Park et al. 2016, 2015). Additionally, centaurone A and centaurein B isolated from AOM demonstrate therapeutic effects against skin cancer in mouse models (Chun et al. 2002). Topical application of AOM methanol extract significantly inhibits 12‐O‐tetradecanoylphorbol‐13‐acetate (TPA)‐induced skin tumor growth and ear edema (Lee et al. 1998). This extract also induces programmed cell death in human leukemia HL‐60 cells, effectively inhibiting their survival and proliferation.

AOE shows protective effects through several reported mechanisms in osteoarthritis treatment. In osteoarthritis models, AOE not only alleviates joint pain but also significantly inhibits cartilage degradation, demonstrating chondroprotective properties (Lee et al. 2019). In IL‐1α‐treated rabbit articular cartilage, AOE inhibits proteoglycan degradation and exhibits significant anti‐inflammatory and analgesic activity (Yu et al. 2020). In lipopolysaccharide‐stimulated RAW 264.7 macrophages, AOE reduces production of key inflammatory mediators including NO, PGE2, IL‐1β, and IL‐6 by suppressing the MAPK signaling pathway (Lee et al. 2019). At the molecular level, AOE plays an important role by regulating the balance between matrix metalloproteinases (MMPs)—responsible for extracellular matrix degradation in osteoarthritis pathogenesis (Loeser 2006), and their inhibitor TIMP‐1, which maintains cartilage homeostasis by inhibiting MMP activity (Tchetverikov et al. 2004). AOE effectively inhibits collagen degradation by modulating this balance (Jeon et al. 2021). Furthermore, AOM water extract (WEAO) inhibits osteoclast differentiation and bone loss by suppressing RANKL‐induced differentiation of bone marrow stromal cells into osteoclasts during early osteoblast differentiation (Ha et al. 2014). Mechanistic studies show that WEAO inhibits the canonical NF‐κB signaling pathway, reduces expression of the upstream activator c‐Fos, and consequently suppresses expression of NFATC1, the master regulator of osteoclast differentiation, without affecting bone resorption activity of mature osteoclasts (Ha et al. 2014).

AOM and its active constituents also demonstrate anti‐allergic properties. WEAO dose‐dependently inhibits histamine release from rat peritoneal mast cells (Kim et al. 2000) and suppresses systemic anaphylaxis (Shin et al. 2001). The active component tectochrysin significantly alleviates shrimp tropomyosin‐induced airway inflammation in mice, manifested as reduced eosinophil infiltration and mucus secretion in lung tissue, while inhibiting Th2 cytokine (IL‐4 and IL‐5) production in bronchoalveolar lavage fluid and enhancing catalase and glutathione peroxidase activities (Fang et al. 2021). These results indicate that AOM coordinately alleviates allergic airway inflammation through multiple pathways: stabilizing mast cells, suppressing Th2 immune responses, and enhancing antioxidant defenses. To facilitate a rapid overview of the reported mechanisms discussed above, the representative compounds, their molecular targets, pathways, and associated disease indications are summarized in (Table 2).

**TABLE 2:** Representative bioactive compounds, extracts, and formulas from AOM, their molecular targets, mechanisms of action, evidence levels, and associated disease indications.

Compound/extract/formula	Type	Molecular target/pathway	Mechanism of action	Disease indication	Evidence level	References
Nootkatone	Single compound	Nrf2/HO‐1, NF‐κB, caspase‐3/9	Antioxidant, anti‐inflammatory, anti‐apoptotic	Diabetic nephropathy, AD	In vivo	Chen et al. (2021); He et al. (2018)
Oxyphyll A	Single compound	Akt/GSK3β, Nrf2/Keap1/HO‐1, PKA/CREB/PGC‐1α	Neuroprotection, mitochondrial biogenesis	AD, PD	In vitro, in vivo	Bian et al. (2021); Shao et al. (2024)
Tectochrysin	Single compound	EGFR, NF‐κB, AP‐1	Antitumor, anti‐inflammatory	Colon cancer, allergic airway inflammation	In vitro, in vivo	Fang et al. (2021); Park et al. (2016, 2015)
Protocatechuic acid (PCA)	Single compound	Nrf2, NF‐κB, mitochondrial permeability	Neuroprotection, antioxidant	AD, PD, cerebral ischemia	In vitro, in vivo	Guan et al. (2006); He et al. (2020); Shi et al. (2006)
p‐Coumaric acid (P‐CA)	Single compound	BDNF/TrkB	Promotes neurogenesis	Cerebral ischemia	In vivo	He et al. (2020)
AOP70‐1	Single compound (polysaccharide)	TLR4/MyD88/NF‐κB	Anti‐neuroinflammation	Neuroinflammation	In vitro, in vivo	Chang et al. (2024)
AOE (extract)	Extract	TLR4, NF‐κB, JNK	Anti‐inflammatory	Cerebral ischemia	In vivo	Cheng et al. (2021)
AOM volatile oil	Extract	TLR4/MyD88/NF‐κB	Antidepressant‐like	Depression	In vivo	Wu et al. (2023)
ASHP (AOM + Schisandra chinensis)	Formula	PI3K/Akt/GSK3β/CREB, NF‐κB	Neuroprotection	AD	In vivo	Qi, Cheng, Gong, et al. 2020; Qi et al. (2019)

Abbreviations: AD, Alzheimer's disease; Akt, protein kinase B; BDNF, brain‐derived neurotrophic factor; COX‐2, cyclooxygenase‐2; GSK3β, glycogen synthase kinase‐3β; HO‐1, heme oxygenase‐1; IL‐6, interleukin‐6; iNOS, inducible nitric oxide synthase; NF‐κB, nuclear factor kappa B; Nrf2, nuclear factor erythroid 2‐related factor 2; PD, Parkinson's disease; PI3K, phosphatidylinositol 3‐kinase; TLR4, Toll‐like receptor 4; TNF‐α, tumor necrosis factor‐α; TrkB, tropomyosin receptor kinase B.

## 5 Translational Challenges and Future Perspectives for AOM‐Based Functional Food Development

The reported pharmacological activities of AOM in neuroprotection, anti‐inflammation, and metabolic regulation provide a compelling scientific rationale for its development as a functional food ingredient (Korsapati et al. 2025). However, translating these preclinical findings into practical applications faces substantial hurdles that must be critically acknowledged. This section examines the key translational barriers, including bioavailability limitations, quality control challenges, and delivery system constraints, and discusses promising technological strategies to address them, while clearly distinguishing between currently feasible approaches and those remaining hypothetical.

### 5.1 Bioavailability and Pharmacokinetic Barriers

A fundamental obstacle to the effective use of AOM bioactive constituents is their poor oral bioavailability. Many phytochemicals, particularly flavonoids and terpenoids, exhibit low aqueous solubility, extensive first‐pass metabolism, and rapid systemic clearance. For example, curcumin, a diarylheptanoid structurally similar to yakuchinone A and B from AOM, has poor oral bioavailability in humans due in part to extensive glucuronidation and sulfation (Anand et al. 2007). Although direct pharmacokinetic data for AOM constituents remain limited, available evidence indicates that AOM extract studies have detected only low or trace plasma levels of parent chemicals after oral administration (Chen et al. 2014). Protocatechuic acid also exhibits relatively low systemic exposure following oral administration, with a short tmax and a relatively short elimination half‐life in rats (Han et al. 2007).

For neuroprotective applications, the blood–brain barrier (BBB) imposes an additional layer of restriction. Even when phytochemicals reach the systemic circulation, their ability to cross the BBB is governed by physicochemical properties such as lipophilicity, molecular weight, and active efflux transporter interactions. Many promising neuroprotective phytochemicals fail to achieve therapeutically relevant concentrations in the central nervous system despite potent in vitro activity. For AOM constituents, systematic data on brain penetration are currently lacking, representing a critical knowledge gap that must be addressed before any rational formulation design can proceed.

### 5.2 Quality Control and Standardization Challenges

The inherent chemical complexity of botanical extracts poses significant challenges for product consistency and reproducibility. AOM extracts contain multiple constituents, and their overall bioactivity cannot yet be attributed to a single validated active principle, making traditional single‐marker quality control inadequate for ensuring product consistency. Variability in cultivation conditions, harvest time, extraction methods, and post‐harvest processing can substantially alter the phytochemical profile and, consequently, the biological activity of AOM preparations. Chromosome‐level genomic and multi‐omics analyses have provided insights into geo‐herbalism‐associated variation in *A. oxyphylla* (Pan et al. 2023). Integrated transcriptome–metabolome profiling further identified nootkatone and kaempferol as quality‐associated biomarkers that distinguish geo‐authentic from non‐authentic regions, and linked regional differences to candidate biosynthetic genes (Pan et al. 2025). These findings support multi‐marker rather than single‐marker standardization. For functional food applications, this variability directly impacts both efficacy and safety; consumers cannot be assured of consistent exposure to the active constituents.

Current regulatory frameworks for botanicals in functional foods remain fragmented globally. Neither specific international standards nor comprehensive national guidance exists for safety and efficacy requirements of functional foods containing botanical ingredients. This regulatory uncertainty complicates any effort to develop AOM‐based products with validated health claims.

### 5.3 Advanced Delivery Systems: Progress and Limitations

To overcome bioavailability and BBB penetration barriers, nanotechnology‐enabled delivery systems have emerged as a promising strategy (Korsapati et al. 2025; Naim et al. 2026). Lipid‐based nanocarriers, including solid lipid nanoparticles, nanostructured lipid carriers, and liposomes, have been shown to improve the physiological stability of phytochemicals, enhance their oral absorption, and facilitate BBB transport (Korsapati et al. 2025). Polymeric nanoparticles and nanoemulsions offer additional formulation options (Korsapati et al. 2025). For instance, curcumin encapsulated in phospholipid complexes or liposomal carriers demonstrated significantly higher brain uptake and antioxidant activity compared with unformulated curcumin in rodent AD models (Naim et al. 2026). Similarly, intranasal administration has emerged as a noninvasive route that bypasses the BBB by directly delivering therapeutics from the nasal cavity to the brain along the olfactory and trigeminal nerve pathways (Korsapati et al. 2025).

However, several limitations must be honestly acknowledged (Korsapati et al. 2025). First, the majority of nano‐delivery studies remain at the preclinical stage, with limited translation to human applications. Second, concerns regarding nanocarrier‐associated toxicity, long‐term safety, and scalability for commercial production remain inadequately addressed. Third, the regulatory pathway for nano‐formulated botanical products is even less defined than for conventional extracts. For AOM specifically, while recent work has explored AOP‐based nanoparticles loaded with nootkatone for acute kidney injury (Deng et al. 2025), systematic investigation of nano‐enabled delivery for neuroprotective AOM constituents has not yet been reported. Therefore, while advanced delivery systems represent a promising direction, their application to AOM‐based neuroprotective products remains hypothetical at this stage and requires dedicated formulation studies, pharmacokinetic profiling, and safety evaluation.

### 5.4 From Preclinical Promise to Clinical Evidence

Perhaps the most critical translational gap is the scarcity of human clinical data. The vast majority of evidence for AOM's neuroprotective and metabolic effects derives from in vitro experiments and rodent models (Saikat and Siam 2026). While these studies provide mechanistic insights and proof‐of‐concept, they cannot substitute for well‐designed human trials. Large‐scale, randomized controlled trials with biomarker‐guided endpoints are essential to validate whether the promising preclinical effects translate to meaningful cognitive or metabolic benefits in specific populations, such as individuals with mild cognitive impairment, prediabetes, or metabolic syndrome.

Currently, clinical data on AOM remain extremely limited. This evidence gap fundamentally limits any claim regarding AOM's efficacy in humans and underscores that all proposed functional food applications remain conceptual rather than evidence‐based.

Importantly, the evidence summarized for AD, PD, depression, diabetes, hyperuricemia, and related indications is predominantly preclinical, consisting mainly of in vitro and rodent studies. Human evidence for AOM as a single agent remains insufficient to support conclusions regarding clinical efficacy; accordingly, disease‐related effects discussed in this review should not be extrapolated to humans without prospective clinical validation.

## 6 Safety, Toxicology, and Regulatory Considerations

Despite the extensive preclinical efficacy data summarized above, the safety profile of AOM and its bioactive constituents remains critically undercharacterized, a gap that is particularly concerning given the manuscript's proposal of AOM as a functional food and nutraceutical ingredient for long‐term consumption.

Traditional Chinese medicine literature characterizes AOM as nontoxic at conventional therapeutic doses; however, systematic acute and repeated‐dose toxicity data for AOM extracts or fruits remain limited. In contrast, a recent GLP‐compliant toxicological evaluation of PD‐00105, a synthetic neuroprotective molecule derived from an AOM constituent (rather than the fruit or a crude extract), revealed dose‐dependent liver and kidney toxicity at high doses (50–100 mg/kg BW/day) in a 90‐day repeated‐dose study (Le Bloch et al. 2024), establishing a NOAEL of 10 mg/kg BW/day and substantiating its use in dietary supplements only at ≤ 10 mg/day in humans.

Pharmacokinetic data are limited to a few terpenoids and herb‐pair formulations in rodents (Qi, Cheng, Jing, et al. 2020), with no human pharmacokinetic data available for any AOM constituent; systematic ADME profiles for major compound classes, including diphenylheptanoids, polysaccharides, and flavonoids—remain entirely lacking. Herb–drug interaction studies are completely absent from the peer‐reviewed literature, and long‐term safety evaluations beyond 90 days have not been conducted, leaving critical questions regarding cumulative toxicity, gut microbiota modulation, and potential withdrawal effects unanswered. Traditional contraindications (e.g., avoidance in Yin deficiency with fire excess) derive from empirical theory rather than modern pharmacological evidence. From a regulatory perspective, the inherent chemical variability of AOM extracts, the absence of international consensus on botanical safety requirements, and the complete lack of human pharmacokinetic and safety data fundamentally preclude evidence‐based approval of health claims for AOM‐based products. Until comprehensive toxicological profiling, systematic pharmacokinetic studies, herb‐drug interaction screening, and long‐term safety evaluations are conducted, all proposals for AOM‐based functional foods, nutraceuticals, or precision nutrition interventions must be interpreted as research hypotheses rather than evidence‐based recommendations for consumption, a caveat that should be explicitly acknowledged alongside the promising preclinical efficacy data summarized in this review.

## 7 Critical Appraisal of Current Evidence: Limitations and Methodological Caveats

The preceding sections have systematically collated the predominantly favorable pharmacological findings of *A. oxyphylla*. However, a balanced scientific assessment demands explicit acknowledgment of the inherent methodological constraints and translational gaps that permeate the current literature. This section critically examines these underappreciated limitations, which collectively temper overoptimistic interpretation and delineate priorities for future confirmatory research.

### 7.1 Heterogeneity in Extract Preparation, Dosage, and Chemical Profiling

A major confounding variable across the cited studies lies in the substantial diversity of extraction protocols; aqueous, ethanolic, ethyl acetate, and hydrodistillation methods yield markedly different chemical profiles, rendering direct cross‐study comparisons problematic. Divergent bioactivities may reflect differential chemical composition rather than genuine pharmacological distinctions. Furthermore, the phytochemical profiles of AOM vary significantly with geographical origin, harvest maturity, and post‐harvest processing, factors frequently inadequately characterized in published reports.

Equally critical is the translational relevance of administered doses. Rodent studies typically use oral gavage dosing, which when converted to human equivalent doses often exceeds amounts reasonably consumed through dietary intake or conventional functional food formulations. This dose disparity raises a fundamental question: do the observed effects represent pharmacological intervention with supra‐physiological concentrations, rather than nutritional modulation achievable through daily consumption? Current literature rarely addresses this distinction, yet it is pivotal for defining realistic application scenarios.

### 7.2 Intrinsic Limitations of Preclinical Models and Over‐Reliance on In Silico Predictions

The evidence base relies heavily on immortalized cell lines, including SH‐SY5Y, PC12, and RAW 264.7 macrophages, subjected to acute chemical insults. While these simplified systems offer valuable mechanistic snapshots, they inadequately recapitulate the chronic, multifactorial, age‐dependent pathophysiology of human diseases. Transgenic rodent models capture certain histopathological hallmarks but do not fully mirror the etiological heterogeneity of sporadic conditions. Species‐specific differences in metabolism, immune reactivity, and neuroanatomy further limit direct extrapolation to humans, and none of the reviewed studies have validated findings in human‐derived three‐dimensional organoid or non‐human primate systems.

Additionally, network pharmacology and molecular docking are extensively employed to predict potential targets of AOM constituents. Although these computational tools generate heuristic hypotheses, they are inherently probabilistic rather than definitive. The majority of cited studies lack orthogonal experimental validation, such as genetic knockdown, pharmacological inhibition with specific antagonists, or direct binding affinity measurements, to confirm that predicted targets are indeed engaged by AOM components in living systems. Consequently, many proposed “mechanisms” remain at the level of bioinformatic inference rather than experimentally substantiated causality.

### 7.3 Publication Bias and the Absence of Negative or Contradictory Findings

It is striking that nearly all retrieved publications report beneficial outcomes, with a conspicuous absence of negative, null, or contradictory results, a pattern strongly suggestive of publication bias. Studies demonstrating statistical significance or positive effects are preferentially published, while investigations yielding equivocal or unfavorable outcomes remain unreported. Furthermore, instances of inter‐batch variability in extract efficacy, non‐monotonic dose–response relationships, or discordance between in vitro potency and in vivo performance are rarely documented in the primary literature. This selective reporting inevitably skews the perceived therapeutic profile of AOM, underestimates its potential ineffectiveness or adverse effects under specific conditions, and compromises the reliability of systematic reviews that depend on complete and unbiased data.

## 8 Conclusion and Future Perspectives

This review summarizes preclinical evidence suggesting that AOM may represent a valuable medicinal and edible resource, with health‐promoting effects observed in preclinical models involving multiple compound classes and biological pathways. Studies have revealed that AOM is rich in diverse bioactive compounds, including diphenylheptanoids, flavonoids, terpenoids, polysaccharides, and phenolic acids, which have been associated with a broad range of pharmacological activities across experimental models. Mechanistically, AOM has been reported to modulate processes relevant to both neural and metabolic homeostasis. On one hand, its neuroprotective effects have been associated with modulation of neuroinflammation and oxidative stress, and the PI3K/Akt pathway has been proposed as a potential integrating node. On the other hand, preclinical studies suggest that AOM may modulate metabolic function by regulating glucose and lipid metabolism, improving insulin sensitivity, and remodeling gut microbiota. Furthermore, some AOM‐containing combinations have shown enhanced effects in preclinical models, along with diverse bioactivities such as cardiovascular protection, antitumor effects, and anti‐allergic properties. These findings suggest potential for further investigation, although clinical validation is needed before any health‐oriented product development.

Despite considerable progress in AOM research, several key challenges and opportunities remain on its path toward modernization and development as high‐value functional foods. Currently, the understanding of the precise molecular targets and in vivo pathways of many AOM constituents, particularly polysaccharides and terpenoids, remains incomplete. Future studies should integrate chemical biology, multi‐omics technologies, and gene‐editing tools to deeply elucidate the compound‐target‐pathway network, with particular emphasis on testing whether interactions among different components are additive, synergistic, or independent. Moreover, as most current evidence is derived from preclinical models, there is an urgent need for well‐designed human clinical trials and epidemiological studies to validate the actual health benefits of AOM in specific populations, such as those with mild cognitive impairment or prediabetes, and to provide robust scientific support for product health claims.

## Author Contributions

**Ziyan Yang:** writing – original draft, formal analysis, investigation. **Hemei Yuan:** investigation, writing – original draft, methodology. **Dengyou Nie:** investigation. **Jun Sheng:** resources, supervision. **Titi Liu:** resources, supervision, project administration, conceptualization. **Huanhuan Xu:** conceptualization, resources, writing – review and editing, funding acquisition.

## Funding

This study was supported by the grants from the Hainan Provincial Key Research and Development Program (ZDYF2025SXLH005), the Xingdian Talent Plan of Yunnan Province (XDYC‐QNRC‐2023‐0427), and the National Natural Science Foundation of China (82404638).

## Disclosure

Author approval and responsibility statement: All authors have read and approved the final version of the manuscript. Titi Liu and Huanhuan Xu had full access to all of the data in this study and take complete responsibility for the integrity of the data and the accuracy of the data analysis.

## Ethics Statement

The authors have nothing to report.

## Conflicts of Interest

The authors declare no conflicts of interest.

## Data Availability

Data sharing not applicable to this article as no datasets were generated or analyzed during the current study.

## References

[fsn372553-bib-0001] Ahmed‐Belkacem, A. , A. Pozza , F. Muñoz‐Martínez , et al. 2005. “Flavonoid Structure‐Activity Studies Identify 6‐Prenylchrysin and Tectochrysin as Potent and Specific Inhibitors of Breast Cancer Resistance Protein ABCG2.” Cancer Research 65, no. 11: 4852–4860. 10.1158/0008-5472.Can-04-1817. 15930306

[fsn372553-bib-0002] An, J. , H. Xiao‐Qiang , L. Shun‐He , et al. 2023. “Regulation of Intestinal Micro Ecology Between Raw and Salt‐Processed *Alpinia oxyphylla* on Renal Injury Rats.” Pakistan Journal of Pharmaceutical Sciences 36, no. 2: 557–564. 10.36721/PJPS.2023.36.2.REG.557-564. 37530165

[fsn372553-bib-0003] Anand, P. , A. B. Kunnumakkara , R. A. Newman , and B. B. Aggarwal . 2007. “Bioavailability of Curcumin: Problems and Promises.” Molecular Pharmaceutics 4, no. 6: 807–818. 10.1021/mp700113r. 17999464

[fsn372553-bib-0004] Bai, W. , T. Wang , X. Yang , Z. Wang , H. Li , and J. Geng . 2025. “Two New Sesquiterpenoids From the Fruits of *Alpinia oxyphylla* .” Natural Product Research 39, no. 3: 468–474. 10.1080/14786419.2023.2272282. 37865974

[fsn372553-bib-0005] Bian, Q. Y. , S. Y. Wang , L. J. Xu , C. O. Chan , D. K. Mok , and S. B. Chen . 2013. “Two New Antioxidant Diarylheptanoids From the Fruits of *Alpinia oxyphylla* .” Journal of Asian Natural Products Research 15, no. 10: 1094–1099. 10.1080/10286020.2013.816297. 23869536

[fsn372553-bib-0006] Bian, Y. , Y. Chen , X. Wang , et al. 2021. “Oxyphylla A Ameliorates Cognitive Deficits and Alleviates Neuropathology via the Akt‐GSK3β and Nrf2‐Keap1‐HO‐1 Pathways In Vitro and In Vivo Murine Models of Alzheimer's Disease.” Journal of Advanced Research 34: 1–12. 10.1016/j.jare.2021.09.002. PMC8655137 35024177

[fsn372553-bib-0007] Breijyeh, Z. , and R. Karaman . 2020. “Comprehensive Review on Alzheimer's Disease: Causes and Treatment.” Molecules 25, no. 24: 5789. 10.3390/molecules25245789. PMC7764106 33302541

[fsn372553-bib-0008] Chan, Y. , W. Chen , Y. Chen , et al. 2020. “Yi‐Zhi‐Fang‐Dai Formula Exerts a Protective Effect on the Injury of Tight Junction Scaffold Proteins In Vitro and In Vivo by Mediating Autophagy Through Regulation of the RAGE/CaMKKβ/AMPK/mTOR Pathway.” Biological & Pharmaceutical Bulletin 43, no. 12: 1847–1858. 10.1248/bpb.b20-00379. 33268702

[fsn372553-bib-0009] Chang, X. , D. Zhang , W. Shi , et al. 2024. “An Arabinoxylan (AOP70‐1) Isolated From Alpinia Oxyphylla Alleviates Neuroinflammation and Neurotoxicity by TLR4/MyD88/NF‐κB Pathway.” International Journal of Biological Macromolecules 277, no. Pt 3: 134339. 10.1016/j.ijbiomac.2024.134339. 39089558

[fsn372553-bib-0010] Chang, Y. M. , H. H. Chang , C. C. Tsai , et al. 2017. “ *Alpinia oxyphylla* Miq. Fruit Extract Activates IGFR‐PI3K/Akt Signaling to Induce Schwann Cell Proliferation and Sciatic Nerve Regeneration.” BMC Complementary and Alternative Medicine 17, no. 1: 184. 10.1186/s12906-017-1695-2. PMC5374583 28359314

[fsn372553-bib-0011] Chang, Y. M. , M. A. Shibu , C. S. Chen , et al. 2021. “Adipose Derived Mesenchymal Stem Cells Along With *Alpinia oxyphylla* Extract Alleviate Mitochondria‐Mediated Cardiac Apoptosis in Aging Models and Cardiac Function in Aging Rats.” Journal of Ethnopharmacology 264: 113297. 10.1016/j.jep.2020.113297. 32841691

[fsn372553-bib-0012] Chang, Y. M. , S. Tamilselvi , H. J. Lin , et al. 2019. “ *Alpinia oxyphylla* Miq Extract Ameliorates Cardiac Fibrosis Associated With D‐Galactose Induced Aging in Rats.” Environmental Toxicology 34, no. 2: 172–178. 10.1002/tox.22671. 30367734

[fsn372553-bib-0013] Chang, Y. M. , C. T. Tsai , C. C. Wang , et al. 2013. “ *Alpinate oxyphyllae* Fructus (Alpinia Oxyphylla Miq) Extracts Inhibit Angiotensin‐II Induced Cardiac Apoptosis in H9c2 Cardiomyoblast Cells.” Bioscience, Biotechnology, and Biochemistry 77, no. 2: 229–234. 10.1271/bbb.120541. 23391902

[fsn372553-bib-0014] Chen, C. M. , C. Y. Lin , Y. P. Chung , et al. 2021. “Protective Effects of Nootkatone on Renal Inflammation, Apoptosis, and Fibrosis in a Unilateral Ureteral Obstructive Mouse Model.” Nutrients 13, no. 11: 3921. 10.3390/nu13113921. PMC8621682 34836176

[fsn372553-bib-0015] Chen, F. , H. L. Li , Y. F. Tan , et al. 2014. “Identification of Known Chemicals and Their Metabolites From *Alpinia oxyphylla* Fruit Extract in Rat Plasma Using Liquid Chromatography/Tandem Mass Spectrometry (LC‐MS/MS) With Selected Reaction Monitoring.” Journal of Pharmaceutical and Biomedical Analysis 97: 166–177. 10.1016/j.jpba.2014.04.037. 24879483

[fsn372553-bib-0016] Chen, T. X. , Shahidin , Q. Q. Zhu , et al. 2026. “Phytochemical Diversity, Mechanistic Pharmacology, and Therapeutic Potential of Alpinia Oxyphylla.” Food 15, no. 7: 1212. 10.3390/foods15071212. PMC13074042 41976506

[fsn372553-bib-0017] Cheng, C. Y. , S. Y. Chiang , S. T. Kao , and S. C. Huang . 2021. “ *Alpinia oxyphylla* Miq Extract Reduces Cerebral Infarction by Downregulating JNK‐Mediated TLR4/T3JAM‐ and ASK1‐Related Inflammatory Signaling in the Acute Phase of Transient Focal Cerebral Ischemia in Rats.” Chinese Medicine 16, no. 1: 82. 10.1186/s13020-021-00495-2. PMC8379872 34419138

[fsn372553-bib-0018] Chun, K. S. , K. K. Park , J. Lee , M. Kang , and Y. J. Surh . 2002. “Inhibition of Mouse Skin Tumor Promotion by Anti‐Inflammatory Diarylheptanoids Derived From *Alpinia oxyphylla* Miquel (Zingiberaceae).” Oncology Research 13, no. 1: 37–45. 10.3727/096504002108747944. 12201673

[fsn372553-bib-0019] Cui, C. , S. L. Wu , J. J. Chen , P. Gongpan , M. Guan , and C. A. Geng . 2023. “Sesquiterpenoids From *Alpinia oxyphylla* With GLP‐1 Stimulative Effects Through ca(2+)/CaMKII and PKA Pathways and Multiple‐Enzyme Inhibition.” Journal of Agricultural and Food Chemistry 71, no. 43: 16148–16159. 10.1021/acs.jafc.3c06093. 37871265

[fsn372553-bib-0020] Dai, C. , M. Liu , Q. Zhang , S. Das Gupta , S. Tang , and J. Shen . 2023. “Nootkatone Supplementation Attenuates Carbon Tetrachloride Exposure‐Induced Nephrotoxicity in Mice.” Antioxidants (Basel) 12, no. 2: 370. 10.3390/antiox12020370. PMC9951873 36829928

[fsn372553-bib-0021] Deng, D. , R. Zhang , J. Wu , et al. 2025. “A Kidney Protection Nanoparticle Based on *Alpinia oxyphylla* Fructus Polysaccharide by Modulating Macrophage Polarization.” International Journal of Biological Macromolecules 292: 139367. 10.1016/j.ijbiomac.2024.139367. 39743078

[fsn372553-bib-0022] Dong, J. , M. Zhou , Q. Qin , et al. 2023. “Structurally Diverse New Eudesmane Sesquiterpenoids With Anti‐Inflammatory Activity From the Fruits of *Alpinia oxyphylla* .” Bioorganic Chemistry 134: 106431. 10.1016/j.bioorg.2023.106431. 36857933

[fsn372553-bib-0023] Du, G. , M. Xiao , X. Zhang , et al. 2017. “ *Alpinia oxyphylla* Miq. Extract Changes miRNA Expression Profiles in db‐/db‐ Mouse Kidney.” Biological Research 50, no. 1: 9. 10.1186/s40659-017-0111-1. PMC5331689 28249617

[fsn372553-bib-0024] Fang, L. , Y. Yan , Z. Xu , et al. 2021. “Tectochrysin Ameliorates Murine Allergic Airway Inflammation by Suppressing Th2 Response and Oxidative Stress.” European Journal of Pharmacology 902: 174100. 10.1016/j.ejphar.2021.174100. 33878335

[fsn372553-bib-0025] Fang, T. , D. Zhou , L. Lu , X. Tong , J. Wu , and L. Yi . 2016. “LXW7 Ameliorates Focal Cerebral Ischemia Injury and Attenuates Inflammatory Responses in Activated Microglia in Rats.” Brazilian Journal of Medical and Biological Research 49, no. 9: e5287. 10.1590/1414-431x20165287. PMC4988477 27533766

[fsn372553-bib-0026] Ge, C. C. , X. Y. Li , W. H. Qiao , et al. 2024. “BACE1 Inhibitors From the Fruits of *Alpinia oxyphylla* Have Efficacy to Treat T2DM‐Related Cognitive Disorder.” Fitoterapia 178: 106157. 10.1016/j.fitote.2024.106157. 39098735

[fsn372553-bib-0027] Guan, S. , B. Jiang , Y. M. Bao , and L. J. An . 2006. “Protocatechuic Acid Suppresses MPP+ −Induced Mitochondrial Dysfunction and Apoptotic Cell Death in PC12 Cells.” Food and Chemical Toxicology 44, no. 10: 1659–1666. 10.1016/j.fct.2006.05.004. 16806628

[fsn372553-bib-0028] Ha, H. , K. S. Shim , T. Kim , et al. 2014. “Water Extract of the Fruits of *Alpinia oxyphylla* Inhibits Osteoclast Differentiation and Bone Loss.” BMC Complementary and Alternative Medicine 14: 352. 10.1186/1472-6882-14-352. PMC4182795 25249312

[fsn372553-bib-0029] Han, Y. , Z. Xiong , C. Yang , M. Liu , and F. Li . 2007. “Determination of Protocatechuic Acid in Rat Plasma by High Performance Liquid Chromatography.” Se Pu = Chinese Journal of Chromatography 25, no. 2: 207–210. 17580687

[fsn372553-bib-0030] He, B. , F. Xu , F. Xiao , et al. 2018. “Neuroprotective Effects of Nootkatone From *Alpiniae oxyphyllae* Fructus Against Amyloid‐β‐Induced Cognitive Impairment.” Metabolic Brain Disease 33, no. 1: 251–259. 10.1007/s11011-017-0154-6. 29177693

[fsn372553-bib-0031] He, B. , F. Xu , T. Yan , et al. 2019. “Tectochrysin From *Alpinia oxyphylla* Miq. Alleviates Aβ(1‐42) Induced Learning and Memory Impairments in Mice.” European Journal of Pharmacology 842: 365–372. 10.1016/j.ejphar.2018.11.002. 30412728

[fsn372553-bib-0032] He, Y. , S. Chen , B. Tsoi , et al. 2020. “ *Alpinia oxyphylla* Miq. and Its Active Compound P‐Coumaric Acid Promote Brain‐Derived Neurotrophic Factor Signaling for Inducing Hippocampal Neurogenesis and Improving Post‐Cerebral Ischemic Spatial Cognitive Functions.” Frontiers in Cell and Developmental Biology 8: 577790. 10.3389/fcell.2020.577790. PMC7849625 33537297

[fsn372553-bib-0033] Jeon, S. Y. , S. H. Yu , B. S. Lee , et al. 2021. “Chondroprotective Effect of *Alpinia oxyphylla* Extract in Experimentally Induced Cartilage Degradation in Rabbit Articular Cartilage Explants.” Journal of Food Biochemistry 45, no. 5: e13713. 10.1111/jfbc.13713. 33818795

[fsn372553-bib-0034] Kim, S. H. , Y. K. Choi , H. J. Jeong , et al. 2000. “Suppression of Immunoglobulin E‐Mediated Anaphylactic Reaction by *Alpinia oxyphylla* in Rats.” Immunopharmacology and Immunotoxicology 22, no. 2: 267–277. 10.3109/08923970009016420. 10952031

[fsn372553-bib-0035] Korsapati, A. , H. R. Korsapati , and V. Peddireddy . 2025. “Neuroprotective Phytochemicals in Functional Foods: Mechanistic Insights, Translational Challenges, and Nano‐Enabled Delivery Strategies.” Trends in Food Science & Technology 165: 105355. 10.1016/j.tifs.2025.105355.

[fsn372553-bib-0036] Le Bloch, J. , D. Jones‐Dias , R. Ferreira , et al. 2024. “Toxicological Evaluation of *Alpinia oxyphylla*‐Derived Molecule (PD‐00105): In Vitro Genotoxicity Studies and 90‐Day Oral Toxicity Study in Rats.” Toxicology Reports 13: 101684. 10.1016/j.toxrep.2024.101684. PMC11261439 39040953

[fsn372553-bib-0037] Lee, E. , K. K. Park , J. M. Lee , et al. 1998. “Suppression of Mouse Skin Tumor Promotion and Induction of Apoptosis in HL‐60 Cells by *Alpinia oxyphylla* Miquel (Zingiberaceae).” Carcinogenesis 19, no. 8: 1377–1381. 10.1093/carcin/19.8.1377. 9744532

[fsn372553-bib-0038] Lee, H. J. , J. S. Kim , J. W. Yoon , H. D. Kim , and J. H. Ryu . 2006. “Suppression of Inducible Nitric Oxide Synthase Expression by Yakuchinones and Their Analogues.” Chemical & Pharmaceutical Bulletin (Tokyo) 54, no. 3: 377–379. 10.1248/cpb.54.377. 16508196

[fsn372553-bib-0039] Lee, Y. M. , E. Son , S. H. Kim , and D. S. Kim . 2019. “Effect of *Alpinia oxyphylla* Extract In Vitro and in a Monosodium Iodoacetate‐Induced Osteoarthritis Rat Model.” Phytomedicine 65: 153095. 10.1016/j.phymed.2019.153095. 31568919

[fsn372553-bib-0040] Li, G. , Z. Zhang , Q. Quan , et al. 2016. “Discovery, Synthesis, and Functional Characterization of a Novel Neuroprotective Natural Product From the Fruit of *Alpinia oxyphylla* for Use in Parkinson's Disease Through LC/MS‐Based Multivariate Data Analysis‐Guided Fractionation.” Journal of Proteome Research 15, no. 8: 2595–2606. 10.1021/acs.jproteome.6b00152. 27246451

[fsn372553-bib-0041] Li, J. , Q. Du , N. Li , S. Du , and Z. Sun . 2021. “Alpiniae Oxyphyllae Fructus and Alzheimer's Disease: An Update and Current Perspective on This Traditional Chinese Medicine.” Biomedicine & Pharmacotherapy 135: 111167. 10.1016/j.biopha.2020.111167. 33383373

[fsn372553-bib-0042] Li, R. , L. Wang , Q. Zhang , et al. 2022. “ *Alpiniae oxyphyllae* Fructus Possesses Neuroprotective Effects on H(2)O(2) Stimulated PC12 Cells via Regulation of the PI3K/Akt Signaling Pathway.” Frontiers in Pharmacology 13: 966348. 10.3389/fphar.2022.966348. PMC9454318 36091821

[fsn372553-bib-0043] Li, W. J. 2021. “Effect of Volatile Oil From Acorus Tatarinowii and *Alpinia oxyphylla* on hCMEC/D3 Cells Injured by Aβ25‐35 and Preparation of Microemulsion [Master's Thesis, Jiangxi University of Traditional Chinese Medicine]. Nanchang.” 10.27180/d.cnki.gjxzc.2021.000486.

[fsn372553-bib-0044] Li, W. J. , S. Xiao , Q. Zheng , et al. 2021. “Mechanism of Volatile Oil From *Alpinia oxyphylla* in Treating Alzheimer's Disease Based on GC‐MS and Network Pharmacology.” Zhongguo Zhong Yao Za Zhi 46, no. 12: 3052–3057. 10.19540/j.cnki.cjcmm.20210301.401. 34467695

[fsn372553-bib-0045] Li, Y. H. , F. Chen , J. F. Wang , Y. Wang , J. Q. Zhang , and T. Guo . 2013. “Analysis of Nine Compounds From *Alpinia oxyphylla* Fruit at Different Harvest Time Using UFLC‐MS/MS and an Extraction Method Optimized by Orthogonal Design.” Chemistry Central Journal 7: 134. 10.1186/1752-153x-7-134. PMC3750461 23915886

[fsn372553-bib-0046] Liao, J. , and X. Zhao . 2024. “Recent Research Progress on the Chemical Constituents, Pharmacology, and Pharmacokinetics of *Alpinae oxyphyllae* Fructus.” Molecules 29, no. 16: 3905. 10.3390/molecules29163905. PMC11357166 39202984

[fsn372553-bib-0047] Lin, H. J. , S. Ramesh , Y. M. Chang , et al. 2021. “D‐Galactose‐Induced Toxicity Associated Senescence Mitigated by *Alpinate oxyphyllae* Fructus Fortified Adipose‐Derived Mesenchymal Stem Cells.” Environmental Toxicology 36, no. 1: 86–94. 10.1002/tox.23014. 32889782

[fsn372553-bib-0048] Loeser, R. F. 2006. “Molecular Mechanisms of Cartilage Destruction: Mechanics, Inflammatory Mediators, and Aging Collide.” Arthritis and Rheumatism 54, no. 5: 1357–1360. 10.1002/art.21813. PMC1774815 16645963

[fsn372553-bib-0049] Naim, A. , A. M. Farooqui , Badruddeen , et al. 2026. “Nanoengineered Phytochemicals Overcome Blood‐Brain Barrier Constraints in Neurodegenerative Disorders.” Frontiers in Neurology 17: 1792829. 10.3389/fneur.2026.1792829. PMC13050714 41948610

[fsn372553-bib-0050] Pan, K. , S. Dai , J. Tian , et al. 2023. “Chromosome‐Level Genome and Multi‐Omics Analyses Provide Insights Into the Geo‐Herbalism Properties of *Alpinia oxyphylla* .” Frontiers in Plant Science 14: 1161257. 10.3389/fpls.2023.1161257. PMC10285302 37360712

[fsn372553-bib-0051] Pan, K. , Y. Qu , J. Liu , et al. 2025. “Integrated Analysis of Transcriptome and Metabolome Reveals the Molecular Basis of Quality Differences in *Alpinia oxyphylla* Miq. From Geo‐Authentic and Non‐Authentic Areas.” Plant Physiology and Biochemistry 222: 109755. 10.1016/j.plaphy.2025.109755. 40073739

[fsn372553-bib-0052] Park, M. H. , J. E. Hong , C. J. Hwang , et al. 2016. “Synergistic Inhibitory Effect of Cetuximab and Tectochrysin on Human Colon Cancer Cell Growth via Inhibition of EGFR Signal.” Archives of Pharmacal Research 39, no. 5: 721–729. 10.1007/s12272-016-0735-7. 27025376

[fsn372553-bib-0053] Park, M. H. , J. E. Hong , E. S. Park , et al. 2015. “Anticancer Effect of Tectochrysin in Colon Cancer Cell via Suppression of NF‐kappaB Activity and Enhancement of Death Receptor Expression.” Molecular Cancer 14: 124. 10.1186/s12943-015-0377-2. PMC4487202 26123287

[fsn372553-bib-0054] Patel, D. K. 2023. “Ethnopharmacological Uses, Pharmacological Activities, and Therapeutic Applications of Tectochrysin in Medicine: An Important Class of Dietary Flavonoid.” Cardiovascular & Hematological Disorders Drug Targets 23, no. 1: 11–20. 10.2174/1871529x23666230726143243. 37496140

[fsn372553-bib-0055] Peng, W. , P. Li , R. Ling , et al. 2022. “Diversity of Volatile Compounds in Ten Varieties of Zingiberaceae.” Molecules 27, no. 2: 565. 10.3390/molecules27020565. PMC8777948 35056880

[fsn372553-bib-0056] Qi, Y. , X. Cheng , G. Gong , et al. 2020. “Synergistic Neuroprotective Effect of Schisandrin and Nootkatone on Regulating Inflammation, Apoptosis and Autophagy via the PI3K/AKT Pathway.” Food & Function 11, no. 3: 2427–2438. 10.1039/c9fo02927c. 32129354

[fsn372553-bib-0057] Qi, Y. , X. Cheng , H. Jing , et al. 2019. “Effect of *Alpinia oxyphylla*‐Schisandra Chinensis Herb Pair on Inflammation and Apoptosis in Alzheimer's Disease Mice Model.” Journal of Ethnopharmacology 237: 28–38. 10.1016/j.jep.2019.03.029. 30880259

[fsn372553-bib-0058] Qi, Y. , X. Cheng , H. Jing , et al. 2020. “Comparative Pharmacokinetic Study of the Components in *Alpinia oxyphylla* Miq.‐Schisandra Chinensis (Turcz.) Baill. Herb Pair and Its Single Herb Between Normal and Alzheimer's Disease Rats by UPLC‐MS/MS.” Journal of Pharmaceutical and Biomedical Analysis 177: 112874. 10.1016/j.jpba.2019.112874. 31542420

[fsn372553-bib-0059] Qi, Y. , H. Jing , X. Cheng , et al. 2020. “ *Alpinia oxyphylla‐*Schisandra Chinensis Herb Pair Alleviates Amyloid‐β Induced Cognitive Deficits via PI3K/Akt/Gsk‐3β/CREB Pathway.” Neuromolecular Medicine 22, no. 3: 370–383. 10.1007/s12017-020-08595-2. 32140977

[fsn372553-bib-0060] Qi, Y. , Q. Zhou , Y. Zhang , J. Deng , R. Li , and X. Zhang . 2024. “Exploring the Active Components and Potential Mechanisms of *Alpiniae oxyphyllae* Fructus in Treating Diabetes Mellitus With Depression by UPLC‐Q‐Exactive Orbitrap/MS, Network Pharmacology and Molecular Docking.” Metabolic Brain Disease 39, no. 6: 1065–1084. 10.1007/s11011-024-01374-z. 38954241

[fsn372553-bib-0061] Qiu, C. , L. Mu , J. Wang , et al. 2023. “Sesquiterpenoids From the Fruits of *Alpinia oxyphylla* Miq. And Their Neuroprotective Effect.” Phytochemistry 211: 113680. 10.1016/j.phytochem.2023.113680. 37084862

[fsn372553-bib-0062] Ren, F. , J. Lin , M. Zhu , et al. 2024. “Polysaccharides From *Alpinia oxyphylla* Fruit Prevent Hyperuricemia by Inhibiting Uric Acid Synthesis, Modulating Intestinal Flora and Reducing Renal Inflammation.” International Journal of Biological Macromolecules 278, no. Pt 2: 134782. 10.1016/j.ijbiomac.2024.134782. 39151857

[fsn372553-bib-0063] Saikat, S. N. , and N. H. Siam . 2026. “Translational Potential of Medicinal Plants for Alzheimer's Disease: Integrated Evidence From Molecular Mechanisms to Preclinical and Clinical Findings.” Natural Products and Bioprospecting 16, no. 1: 75. 10.1007/s13659-026-00621-3. PMC13338004 42406197

[fsn372553-bib-0064] Schirinzi, T. , F. Di Lorenzo , G. M. Sancesario , et al. 2018. “Amyloid‐Mediated Cholinergic Dysfunction in Motor Impairment Related to Alzheimer's Disease.” Journal of Alzheimer's Disease 64, no. 2: 525–532. 10.3233/jad-171166. 29914023

[fsn372553-bib-0065] Shao, M. , C. Zhao , Z. Pan , et al. 2024. “Oxyphylla A Exerts Antiparkinsonian Effects by Ameliorating 6‐OHDA‐Induced Mitochondrial Dysfunction and Dyskinesia In Vitro and In Vivo.” Chemico‐Biological Interactions 403: 111224. 10.1016/j.cbi.2024.111224. 39233265

[fsn372553-bib-0066] Shi, G. F. , L. J. An , B. Jiang , S. Guan , and Y. M. Bao . 2006. “Alpinia Protocatechuic Acid Protects Against Oxidative Damage In Vitro and Reduces Oxidative Stress In Vivo.” Neuroscience Letters 403, no. 3: 206–210. 10.1016/j.neulet.2006.02.057. 16806694

[fsn372553-bib-0067] Shi, W. , J. Zhong , Q. Zhang , and C. Yan . 2020. “Structural Characterization and Antineuroinflammatory Activity of a Novel Heteropolysaccharide Obtained From the Fruits of *Alpinia oxyphylla* .” Carbohydrate Polymers 229: 115405. 10.1016/j.carbpol.2019.115405. 31826414

[fsn372553-bib-0068] Shin, T. Y. , J. H. Won , H. M. Kim , and S. H. Kim . 2001. “Effect of *Alpinia oxyphylla* Fruit Extract on Compound 48/80‐Induced Anaphylactic Reactions.” American Journal of Chinese Medicine 29, no. 2: 293–302. 10.1142/s0192415x01000319. 11527071

[fsn372553-bib-0069] Sun, Z. , Y. Zhang , M. Zhang , et al. 2023. “Integrated Brain and Plasma Dual‐Channel Metabolomics to Explore the Treatment Effects of *Alpinia oxyphylla* Fructus on Alzheimer's Disease.” PLoS One 18, no. 8: e0285401. 10.1371/journal.pone.0285401. PMC10409282 37552694

[fsn372553-bib-0070] Sung, Y. Y. , and D. S. Kim . 2022. “Synergistic Impacts of *Alpinia oxyphylla* Seed Extract and Allopurinol Against Experimental Hyperuricemia.” BioMed Research International 2022: 2824535. 10.1155/2022/2824535. PMC9206559 35726318

[fsn372553-bib-0071] Sur, B. , S. Kang , M. Kim , and S. Oh . 2019. “Inhibition of Carrageenan/Kaolin‐Induced Arthritis in Rats and of Inflammatory Cytokine Expressions in Human IL‐1β‐Stimulated Fibroblast‐Like Synoviocytes by a Benzylideneacetophenone Derivative.” Inflammation 42, no. 3: 928–936. 10.1007/s10753-018-0947-8. PMC6527524 30565030

[fsn372553-bib-0072] Tchetverikov, I. , H. K. Ronday , B. Van El , et al. 2004. “MMP Profile in Paired Serum and Synovial Fluid Samples of Patients With Rheumatoid Arthritis.” Annals of the Rheumatic Diseases 63, no. 7: 881–883. 10.1136/ard.2003.013243. PMC1755080 15194590

[fsn372553-bib-0073] Wang, Q. , X. Xue , Z. Chen , et al. 2025. “Nootkatone Inhibits the Progression of Glioblastoma by Activating the ATF4‐CHOP‐CHAC1 Pathway.” Molecular Medicine 31, no. 1: 13. 10.1186/s10020-025-01064-1. PMC11737244 39819316

[fsn372553-bib-0074] Wang, R. , X. Ruan , J. Chen , et al. 2024. “Physicochemical Characterization and Biological Properties of Polysaccharides From *Alpiniae oxyphyllae* Fructus.” Polymers (Basel) 16, no. 12: 1705. 10.3390/polym16121705. PMC11207487 38932054

[fsn372553-bib-0075] Wang, S. , Y. Zhao , J. Zhang , et al. 2015. “Antidiarrheal Effect of *Alpinia oxyphylla* Miq. (Zingiberaceae) in Experimental Mice and Its Possible Mechanism of Action.” Journal of Ethnopharmacology 168: 182–190. 10.1016/j.jep.2015.03.066. 25861952

[fsn372553-bib-0076] Wang, Y. , M. Wang , K. Fan , et al. 2018. “Protective Effects of *Alpinae oxyphyllae* Fructus Extracts on Lipopolysaccharide‐Induced Animal Model of Alzheimer's Disease.” Journal of Ethnopharmacology 217: 98–106. 10.1016/j.jep.2018.02.015. 29447949

[fsn372553-bib-0077] Wei, Y. , K. Gao , Y. Sun , Q. Sheng , and J. Guo . 2025. “Neuroprotective Effects and Mechanisms of *Alpiniae oxyphyllae* Fructus, a Medicinal and Edible Homologous Herb: Research Advances.” International Journal of Molecular Sciences 26, no. 13: 6230. 10.3390/ijms26136230. PMC12250459 40650004

[fsn372553-bib-0078] Wu, B. , A. Gan , R. Wang , F. Lin , T. Yan , and Y. Jia . 2023. “ *Alpinia oxyphylla* Miq. Volatile Oil Ameliorates Depressive Behaviors and Inhibits Neuroinflammation in CUMS‐Exposed Mice by Inhibiting the TLR4‐Medicated MyD88/NF‐κB Signaling Pathway.” Journal of Chemical Neuroanatomy 130: 102270. 10.1016/j.jchemneu.2023.102270. 37001682

[fsn372553-bib-0079] Xie, Y. , M. Xiao , D. Li , et al. 2017. “Anti‐Diabetic Effect of *Alpinia oxyphylla* Extract on 57BL/KsJ db‐/db‐ Mice.” Experimental and Therapeutic Medicine 13, no. 4: 1321–1328. 10.3892/etm.2017.4152. PMC5377374 28413472

[fsn372553-bib-0080] Xie, Y. , M. Xiao , Y. Ni , et al. 2018. “ *Alpinia oxyphylla* Miq. Extract Prevents Diabetes in Mice by Modulating Gut Microbiota.” Journal of Diabetes Research 2018: 4230590. 10.1155/2018/4230590. PMC6008625 29967794

[fsn372553-bib-0081] Xu, J. , F. Wang , J. Guo , et al. 2020. “Pharmacological Mechanisms Underlying the Neuroprotective Effects of *Alpinia oxyphylla* Miq. On Alzheimer's Disease.” International Journal of Molecular Sciences 21, no. 6: 2071. 10.3390/ijms21062071. PMC7139528 32197305

[fsn372553-bib-0082] Yan, T. , B. Wu , Z. Z. Liao , et al. 2016. “Brain‐Derived Neurotrophic Factor Signaling Mediates the Antidepressant‐Like Effect of the Total Flavonoids of *Alpiniae oxyphyllae* Fructus in Chronic Unpredictable Mild Stress Mice.” Phytotherapy Research 30, no. 9: 1493–1502. 10.1002/ptr.5651. 27191791

[fsn372553-bib-0083] Yan, Z. , L. Zhang , Y. Kang , et al. 2024. “Integrating Serum Pharmacochemistry and Network Pharmacology to Explore Potential Compounds and Mechanisms of *Alpiniae oxyphyllae* Fructus in the Treatment of Cellular Senescence in Diabetic Kidney Disease.” Frontiers in Medicine (Lausanne) 11: 1424644. 10.3389/fmed.2024.1424644. PMC11251962 39021818

[fsn372553-bib-0084] Yang, X. , Y. Yang , H. Chen , et al. 2020. “Extraction, Isolation, Immunoregulatory Activity, and Characterization of *Alpiniae oxyphyllae* Fructus Polysaccharides.” International Journal of Biological Macromolecules 155: 927–937. 10.1016/j.ijbiomac.2019.11.051. 31712145

[fsn372553-bib-0085] Yong, Z. , H. Zibao , Z. Zhi , et al. 2022. “Nootkatone, a Sesquiterpene Ketone From *Alpiniae oxyphyllae* Fructus, Ameliorates Metabolic‐Associated Fatty Liver by Regulating AMPK and MAPK Signaling.” Frontiers in Pharmacology 13: 909280. 10.3389/fphar.2022.909280. PMC9294377 35865940

[fsn372553-bib-0086] Yu, S. H. , H. J. Kim , S. Y. Jeon , et al. 2020. “Anti‐Inflammatory and Anti‐Nociceptive Activities of *Alpinia oxyphylla* Miquel Extracts in Animal Models.” Journal of Ethnopharmacology 260: 112985. 10.1016/j.jep.2020.112985. 32439403

[fsn372553-bib-0087] Zeng, P. , Y. C. Liu , X. M. Wang , et al. 2022. “Targets and Mechanisms of *Alpinia oxyphylla* Miquel Fruits in Treating Neurodegenerative Dementia.” Frontiers in Aging Neuroscience 14: 1013891. 10.3389/fnagi.2022.1013891. PMC9749063 36533181

[fsn372553-bib-0088] Zhang, Q. , X. Hu , F. Hui , et al. 2017. “Ethanol Extract and Its Dichloromethane Fraction of *Alpinia oxyphylla* Miquel Exhibited Hepatoprotective Effects Against CCl(4)‐Induced Oxidative Damage In Vitro and In Vivo With the Involvement of Nrf2.” Biomedicine & Pharmacotherapy 91: 812–822. 10.1016/j.biopha.2017.04.131. 28501008

[fsn372553-bib-0089] Zhang, Q. , Y. Zheng , X. Hu , et al. 2018. “Ethnopharmacological Uses, Phytochemistry, Biological Activities, and Therapeutic Applications of *Alpinia oxyphylla* Miquel: A Review.” Journal of Ethnopharmacology 224: 149–168. 10.1016/j.jep.2018.05.002. 29738847

[fsn372553-bib-0090] Zhang, S. , Y. Yang , R. Zhang , et al. 2024. “The Potential Mechanism of *Alpiniae oxyphyllae* Fructus Against Hyperuricemia: An Integration of Network Pharmacology, Molecular Docking, Molecular Dynamics Simulation, and In Vitro Experiments.” Nutrients 17, no. 1: 71. 10.3390/nu17010071. PMC11723258 39796505

[fsn372553-bib-0091] Zhang, T. , J. Qiu , X. Wu , S. Huang , H. Yuan , and S. Park . 2020. “Schizonepeta Tenuifolia With *Alpinia oxyphylla* Alleviates Atopic Dermatitis and Improves the Gut Microbiome in Nc/Nga Mice.” Pharmaceutics 12, no. 8: 722. 10.3390/pharmaceutics12080722. PMC7465453 32751987

[fsn372553-bib-0092] Zhang, W. , J. Song , W. Li , et al. 2020. “Salvianolic Acid D Alleviates Cerebral Ischemia‐Reperfusion Injury by Suppressing the Cytoplasmic Translocation and Release of HMGB1‐Triggered NF‐κB Activation to Inhibit Inflammatory Response.” Mediators of Inflammation 2020: 9049614. 10.1155/2020/9049614. PMC7204335 32410871

[fsn372553-bib-0093] Zhang, Z. , G. Li , S. S. W. Szeto , et al. 2015. “Examining the Neuroprotective Effects of Protocatechuic Acid and Chrysin on In Vitro and In Vivo Models of Parkinson Disease.” Free Radical Biology & Medicine 84: 331–343. 10.1016/j.freeradbiomed.2015.02.030. 25769424

[fsn372553-bib-0094] Zhao, J. , D. Ji , Q. Chen , et al. 2024. “Identification of a New Flavonol Glucuronide From *Alpinia oxyphylla* .” Natural Product Research 39: 1–4. 10.1080/14786419.2024.2386129. 39078886

[fsn372553-bib-0095] Zhou, S. , L. Liu , Y. Zhang , et al. 2022. “Integrated Untargeted and Targeted Metabolomics to Reveal Therapeutic Effect and Mechanism of *Alpiniae oxyphyllae* Fructus on Alzheimer's Disease in APP/PS1 Mice.” Frontiers in Pharmacology 13: 1104954. 10.3389/fphar.2022.1104954. PMC9873993 36712678

[fsn372553-bib-0096] Zhu, Y. T. , H. B. Fang , X. N. Liu , et al. 2023. “Unusual Acetylated Flavonol Glucuronides, Oxyphyllvonides A‐H With Renoprotective Activities From the Fruits of *Alpinae oxyphylla* .” Phytochemistry 215: 113849. 10.1016/j.phytochem.2023.113849. 37673290

[fsn372553-bib-0097] Zhu, Y. T. , X. N. Liu , B. T. Lu , Y. X. Cheng , and Y. Z. Wang . 2024. “Novel Sesquiterpenoids With Renoprotective Activities From the Fruits of *Alpinae oxyphylla* as Potent TGF‐β1/Smads Phosphorylation Inhibitors.” Journal of Agricultural and Food Chemistry 72, no. 23: 13138–13153. 10.1021/acs.jafc.4c01720. 38814319

[fsn372553-bib-0098] Zuo, L. , J. Li , L. Xue , et al. 2021. “Integrated UPLC‐MS/MS and UHPLC‐Q‐Orbitrap HRMS Analysis to Reveal Pharmacokinetics and Metabolism of Five Terpenoids From *Alpiniae oxyphyllae* Fructus in Rats.” Current Drug Metabolism 22, no. 1: 70–82. 10.2174/1389200221666201217154219. 33334283

